# English Word and Pseudoword Spellings and Phonological Awareness: Detailed Comparisons From Three L1 Writing Systems

**DOI:** 10.3389/fpsyg.2020.01309

**Published:** 2020-07-02

**Authors:** Katherine I. Martin, Emily Lawson, Kathryn Carpenter, Elisa Hummer

**Affiliations:** ^1^Department of Linguistics, Southern Illinois University, Carbondale, IL, United States; ^2^Center for English as a Second Language, Southern Illinois University, Carbondale, IL, United States; ^3^Department of Curriculum and Instruction, Southern Illinois University, Carbondale, IL, United States

**Keywords:** spelling, phonological awareness, ESL, cross-linguistic influence, abjad, morphosyllabary, alphabet

## Abstract

Spelling is a fundamental literacy skill facilitating word recognition and thus higher-level reading abilities via its support for efficient text processing (Adams, 1990; Joshi et al., 2008; Perfetti and Stafura, 2014). However, relatively little work examines second language (L2) spelling in adults, and even less work examines learners from different first language (L1) writing systems. This is despite the fact that the influence of L1 writing system on L2 literacy skills is well documented (Hudson, 2007; Koda and Zehler, 2008; Grabe, 2009). To address this shortcoming, this study collected data on real word spelling, pseudoword spelling, and phonological awareness (elision) abilities from 70 participants (23 native speakers; 47 ELLs with alphabetic, abjad, and morphosyllabic L1s). Analyses compared performance on real word and pseudoword spelling between L1 English speakers and ELLs, and additionally among the non-native-speaker L1 groups (categorized into alphabet, abjad, and morphosyllabary groups). Similar comparisons were made across groups for performance on phonological awareness. Further, correlations were calculated between phonological awareness and real word spelling and between phonological awareness and pseudoword spelling, separately for L1 English speakers and the various ESL groups. Spelling accuracy on real words and pseudowords as well as phonological awareness skill differed between native speakers and ESL speakers, and also varied by the ESL speakers’ L1 writing system. Theoretically interesting patterns emerged in the spelling data. For example, the morphosyllabic L1 speakers had strong real word spelling (better than the other ESL groups) but greatly decreased pseudoword accuracy (a drop of 59% in accuracy). Although alphabetic L1 speakers had low spelling accuracy in terms of strict scoring, they had lower rates of errors per item, highlighting the importance of scoring approach for shaping the conclusions that are drawn. Error rates also revealed vowels to be more problematic than consonants, particularly in abjad L1 speakers. The results demonstrate that L2 spelling abilities, phonological awareness, and the relationships among them vary by L1 writing system, and that differing approaches to scoring and analysis may lead to varying conclusions.

## Introduction

Literacy is widely recognized as a foundational educational skill (e.g., Snow and Strucker, 1999; Gomez and Gomez, 2007). Although it has received somewhat less attention than other critical literacy skills such as phonological awareness, decoding, and word recognition (Treiman, 1997; Joshi et al., 2008), a number of models of first language (L1) spelling development have been articulated (e.g., Chall, 1983; Frith, 1985; Ehri, 1989; Nunes et al., 1997); the relationships between spelling ability and other components of literacy have been examined (Gill, 1989; de Manrique and Signorini, 1994; MacDonald and Cornwall, 1995; Yeung et al., 2011; Graham and Santangelo, 2014), and best approaches for spelling instruction have been established (e.g., Graham, 1999; Moats, 2005; Joshi et al., 2008; Bear et al., 2019).

In contrast, spelling ability has received relatively little attention in second language (L2) learners. Existing research shows evidence both for overlap with L1 spelling (Wade-Woolley and Siegel, 1997; Durgunoğlu, 1998; Figueredo, 2006; Jongejan et al., 2007) and L1-based differences (Koda, 2004, 2008; Dixon et al., 2010; Dich and Pedersen, 2013). In particular, L2 spelling is influenced by the characteristics of learners’ L1 writing system (Ziegler and Goswami, 2005; Koda and Zehler, 2008; Frost, 2012; Perfetti and Verhoeven, 2017). However, much of this research has focused on young bilingual students, not adult L2 users, and cross-linguistic comparisons with the same tasks and materials are limited. Further, little is known about how L2 spelling ability relates to other L2 literacy skills, and how these relationships vary in learners from different L1s.

To address these gaps, the current study analyzed English spelling data from adult ESL users in three L1 groups (based on L1 writing system: alphabet, abjad, or morphosyllabary), as well as a native speaker comparison group. Data were additionally collected on phonological awareness ability to facilitate cross-linguistic comparisons of not only spelling ability, but also how spelling is related to other key literacy skills.

## Spelling as a Central Component of Literacy

Although spelling ability frequently gets only secondary attention in literacy research (Treiman, 1997; Joshi et al., 2008), it is in fact strongly interrelated with overall literacy skills. Rather than being simply a peripheral, rote, memory-based skill, spelling is fundamentally connected to overall reading and writing abilities (Ehri and Wilce, 1987; Ehri, 1997, 2000; Joshi et al., 2008). Much of this connection is due to the fact that both word recognition and spelling ability draw on the same underlying lexical representations (Snow et al., 2005; Russak and Kahn-Horwitz, 2015). Thus, stable and fully-specified spellings (orthographic forms) within lexical representations support rapid, automatic word recognition during fluent reading (Perfetti and Hart, 2002; Ehri and Snowling, 2004; Perfetti, 2007). Without such robust lexical representations, additional cognitive resources must be allocated to decoding and bottom-up word recognition and retrieval, thus limiting the cognitive resources that can be dedicated to higher-level reading skills such as text integration and inferencing (Ehri, 2005; Mehta et al., 2005; Perfetti, 2007).

Spelling is also a key component of writing. The Simple View of Writing (e.g., Berninger et al., 2002; Berninger and Amtmann, 2003) describes four components of writing skill: transcription (including handwriting and spelling), executive functions (including attention, planning, and reviewing), working memory, and text generation. If one component, such as transcription (i.e., spelling), requires extra attentional resources, the cognitive capacity available for the other components of writing decreases (Wollscheid et al., 2016). In fact, students with poor spelling skills typically write less, with more restricted vocabulary, and at an overall lower level of quality than students with good spelling skills (Graham et al., 1997; Singer and Bashir, 2004; Moats et al., 2006; Re et al., 2007).

Further evidence for the interconnectedness of spelling with word recognition, reading comprehension, and writing comes from research examining the impact of instructional interventions. Targeted spelling instruction improves not only spelling and phonological awareness (e.g., Graham et al., 2002; Berninger and Amtmann, 2003; Graham and Santangelo, 2014) but also word recognition (Post et al., 2001; Graham et al., 2002; Graham and Hebert, 2011), reading fluency (Graham and Hebert, 2011), and writing fluency (Graham et al., 2002). Thus, spelling ability provides a window onto the underlying lexical representations that serve as a foundation for readers’ general literacy skills, both productive and receptive, and can also provide an avenue for improving overall literacy.

One perennial point of contention in literacy research is whether word recognition is best modeled via a single-route, connectionist approach (e.g., Seidenberg and McClelland, 1989; Plaut et al., 1996) or a dual-route approach, with one route progressing via assembling grapheme-phoneme correspondences and one progressing via direct lexical look-up (e.g., Coltheart et al., 1993, 2001). However, this debate is not limited to word recognition. Spelling ability may also draw on multiple component skills, including phonological awareness, recoding, and multiple aspects of orthographic knowledge. These can include knowledge of both whole-word orthographic forms, also known as mental graphemic representations (Apel, 2011) or lexical orthographic knowledge (e.g., Deacon et al., 2012; Conrad et al., 2013; Apel et al., 2019); as well as restrictions on allowable letter combinations and placements, also called graphotactics (e.g., Verhoeven et al., 2006; Deacon et al., 2008), orthotactics (e.g., Masterson and Apel, 2007; Georgiou et al., 2009), or sublexical orthographic knowledge (e.g., Cunningham et al., 2001; Loveall et al., 2013).

Cross-linguistic research on spelling development suggests that the degree to which individuals rely on these two major sources of knowledge – phonological and visual/orthographic – varies across languages, and in particular across writing systems. As has been widely discussed in the literature on cross-linguistic literacy, the varying phonological and morphological characteristics of different languages are typically associated with particular approaches to encoding the language in writing, such that “every language gets the writing system it deserves” (Frost, 2012, p. 266).

Although there are different approaches to classifying writing systems (e.g., compare Daniels and Bright, 1996; Perfetti and Dunlap, 2008; Sampson, 2015), a relatively comprehensive approach uses five general categories: alphabet, abugida, abjad, syllabary, and morphosyllabary. An alphabet is a writing system in which all sounds – both consonants and vowels – have full, independent graphic forms representing them. Languages such as Spanish, Russian, and Greek use alphabets. An abugida (or alphasyllabary) is similar to an alphabet in that it is a segmental system (each spoken sound is represented by a distinct graphic form); however, it differs in that the main forms represent consonants. Vowels are also written but are generally represented by small diacritics or modifications to the main consonant graphs. Many Brahmic languages (including Hindi, Marathi, Nepali, Sanskrit, and Thai), Ethiopic languages (including Ge’ez, Amharic, and Harari), and Cree languages (including Ojibwe and Algonquian) use abugidas. An abjad is similar to an abugida in that it is a consonant-central writing system, with vowels written as diacritics above or below the main line of text; the primary difference is that these vowel graphs are *optional* in abjads, and in fact are frequently omitted in texts written for fluent adult readers (e.g., Share and Levin, 1999; Abu-Rabia, 2001, 2002; Frost, 2006). Arabic and Hebrew are both written with an abjad. In a syllabary, each graph represents a distinct combination of consonant(s) and vowel(s) (a syllable), with additional graphs available for syllable codas as relevant. Japanese uses three different writing systems, two of them (hiragana and katakana) with syllabaries; Cherokee, Vai, and the Yi languages of China also use syllabaries. Finally, in a morphosyllabary (or logography) the written graphs represent whole morphemes, the smallest meaningful unit of language. Similar to a syllabary, morphemes are typically monosyllabic in such languages, meaning that the written graphs correspond to single spoken syllables. However, in a syllabary the number of graphemes is limited to the number of spoken syllables and there is an established relationship between written and spoken forms. In contrast, in a morphosyllabary, there are many more graphemes than spoken syllables, and written forms typically indicate only minimal information about pronunciation. Chinese languages such as Mandarin and Cantonese use a morphosyllabary.

Using a similar classification scheme, Frost (2012, pp. 267–270) describes a variety of ways in which the linguistic characteristics of languages (e.g., phonological limitations on syllable structures, the number of possible syllables, the richness of morphological paradigms) have resulted in pairings between spoken and written language systems with optimal information encoding. For example, in Mandarin and Cantonese, most words comprise only one morpheme (with very little inflection or derivation), most morphemes are monosyllabic, and syllables are typically restricted to four or fewer sounds. This results in a very small number of spoken syllables, and thus a large number of homophones (Chao, 1968). Although many homophones can be distinguished phonetically by tone, the use of additional graphs indicating semantics (i.e., semantic radicals) must be used in written text to disambiguate meaning – pushing Mandarin and Cantonese toward the use of a morphosyllabic writing system (DeFrancis, 1989; Wang et al., 2009). In contrast, Finnish makes extensive use of derivational and inflectional suffixes, resulting in words that are frequently 14 letters or longer (e.g., *kirjastokortti* ‘library card,’ *perhetapahtuma* ‘family event’) (Kuperman et al., 2008). However, it uses a highly regular (i.e., shallow) alphabet, with near-perfect consistency between graphemes and phonemes; this high degree of regularity is necessary for such long, morphologically complex words to be read effectively.

In conjunction with these linguistic variations in languages using different writing systems, the approaches to text processing that develop in fluent readers of different languages are strongly influenced by such L1 characteristics (e.g., Ziegler and Goswami, 2005; Tolchinsky et al., 2012). For example, in shallow writing systems such as German or Spanish, knowledge of phoneme-grapheme correspondences and phonological segmentation abilities are sufficient to accurately spell or read most words (e.g., Frith et al., 1998; Ziegler and Goswami, 2005). However, in deeper orthographies such as English, readers and spellers must have knowledge not only of single sound-form mappings but also more complex mappings between phonological units of varying sizes (e.g., phonemes, rhymes, and syllables) and orthographic sequences of varying lengths (e.g., single letters, digraphs such as < th > or < ck >, non-linear spelling patterns such as VCe, e.g., *hope*, and rimes such as *-ough*) (Goswami et al., 1998; Ziegler and Goswami, 2005).

Research has also indicated that individual spellers may take somewhat different approaches to spelling – either lexical (whole-word) or sub-lexical (based on phoneme-grapheme correspondences) (e.g., Baron, 1979; Treiman, 1984; Castles et al., 1997). These individual tendencies for spelling strategies also pattern with reading skills. For example, Lennox and Siegel (1993, 1996) examined the types of spelling errors produced by children with different profiles of reading and spelling skills. They found that children with reading disabilities and other poor spellers tended to produce less phonologically accurate misspellings and relied on phonological correspondence information to spell words less frequently than normally achieving children and good spellers (see also Bruck and Treiman, 1990; Lennox and Siegel, 1998; Cassar and Treiman, 2004). Thus, literacy research has established the impact of L1 linguistic structure, L1 writing system, and individual differences in overall literacy skills on individuals’ spelling abilities.

## Understanding L2 Spelling

Given the importance of high-quality lexical representations to support word recognition and overall reading skill, surprisingly few studies have included a detailed consideration of L2 spelling skills. This is true even for English as a second/foreign language (ESL/EFL), despite the international prevalence of this second language (e.g., Crystal, 2003). However, the broad themes and findings that have been established are described below to provide a framework for ESL spelling as examined in the present study.

A number of studies on ESL spelling have found substantial overlap between L1 (English) spelling development and L2 (ESL/EFL) spelling development. For example, ESL spelling ability relies on largely the same component literacy skills (e.g., phonological awareness, orthographic knowledge, auditory perception) as does monolingual L1 English spelling ability (Wade-Woolley and Siegel, 1997; Durgunoğlu, 1998; Figueredo, 2006; Jongejan et al., 2007; Russak and Kahn-Horwitz, 2015). In addition, L2 spellers may, in principle, rely on both or either of phonological and orthographic knowledge for spelling in L2. However, research on L2 text processing and spelling suggests that the type of knowledge used most frequently varies based on learners’ L1 background and the literacy processes that develop as attuned to their L1 language and writing system. In other words, the L1-specific text processing approaches that develop along with initial literacy acquisition frequently transfer to and influence L2 text processing approaches (Durgunoğlu, 2002; Wang et al., 2003; Koda and Zehler, 2008). More specifically, learners from shallow, alphabetic L1 backgrounds tend to rely more on phonological skills and decoding/recoding for word-level literacy (including word recognition and spelling), whereas learners from deeper, opaque, and non-alphabetic L1 backgrounds tend to rely more on visual and orthographic information for the same literacy skills (e.g., Holm and Dodd, 1996; Nassaji and Geva, 1999; Wade-Woolley, 1999; Akamatsu, 2003; McBride-Chang et al., 2004; Mayer et al., 2007; Martin and Juffs, in press).

These general tendencies also influence the types of spelling errors or misidentifications that are made most frequently by learners from different L1 backgrounds. For example, Wang and colleagues (Wang et al., 2003; Wang and Koda, 2005) examined the word identification and phonological awareness skills of university-level L1 Chinese and L1 Korean ESL students. They found that the L1 Korean speakers had greater accuracy on a word naming task (dependent on phonological skills) and had more frequent regularization errors in their pronunciations of irregular words than the L1 Chinese speakers. On the other hand, the L1 Chinese speakers were more accurate in a semantic categorization task involving differently spelled homophones (demonstrating an ability to rely on orthographic over phonological form) but had more difficulty making judgments to words with similar spellings. Similarly, in work examining L1 English and L1 Chinese learners of L2 Japanese, Li and Martin (Li and Martin, 2017; Martin and Li, 2017) found that L1 Chinese speakers produced substantially fewer errors overall, and that the types of orthographic errors produced revealed L1 influence. For example, the L1 English speakers tended to produce phonologically based errors, in particular errors resulting from maintaining the English (L1) pronunciation of words borrowed into Japanese, whereas the L1 Chinese speakers tended to transfer in (simplified) Chinese characters in place of the target Japanese kanji characters.

Another well-established domain of L1 influence on L2 spelling is L1 phonology: when examining the specific misspellings produced by L2 spellers, errors frequently appear for the spellings of sounds that do not exist or are not contrastive in learners’ L1. For example, Wang and Geva (2003a, b) examined the ability of L1 Cantonese-speaking children to spell words with /θ/ < th > and /ʃ/ < sh > – phonemes not present in Cantonese. The L1 Cantonese speakers had substantially more errors than matched L1 English speakers in spelling these non-native phonemes as well as lower overall pseudoword spelling accuracy, though they had stronger overall performance on a confrontation spelling task (dependent on orthographic knowledge).

The two groups with the most available research demonstrating an effect of L1 phonology on L2 spelling are L1 Spanish speakers (Cronnell, 1985; Zutell and Allen, 1988; Ferroli and Shanahan, 1992; Fashola et al., 1996; Cook, 1997) and L1 Arabic speakers (Ibrahim, 1978; Cook, 1997; Fender, 2008; Allaith and Joshi, 2011; Saigh and Schmitt, 2012). In Spanish, for example, spellers have been found to frequently confuse < b > and < v > (e.g., < bery > for *very*) and < s > and < z > (e.g., < eazy > for *easy*), as well as the spellings of /i/ and /I/ (e.g., < it > for *eat*). Similarly, in L1 Arabic speakers, spelling errors frequently involve confusion between graphemes representing sounds present in Arabic (e.g., /b/ and /f/) and their voicing pairs, representing sounds absent from Modern Standard Arabic (e.g., /p/ and /v/); examples include < cabable > for *capable* and < habet > for *habit*. Similar evidence of L1 phonological influence on L2 spelling has been documented for L1 German (James and Klein, 1994) and L1 Japanese (Cook, 1997).

Another area of L1 influence on L2 spelling, and L2 text processing in general, is the differential levels of attention that readers and spellers from some L1s have for vowels versus consonants. More specifically, individuals from an abjad L1 (e.g., Arabic, Hebrew) show less awareness of, lower sensitivity to, or less robust representations of vowels compared to consonants – “vowel blindness” (Ryan and Meara, 1996). Early studies with L1 Arabic speakers showed that they process Roman letters much differently from L1 English speakers (Randall and Meara, 1988) and have reduced awareness of vowel letters in English words (Ryan and Meara, 1991; partially replicated by Hayes-Harb, 2006). More recent studies have shown that L1 Arabic speakers use only minimal phonological information in text processing (Fender, 2003; Martin and Juffs, in press). In terms of spelling ability, L1 Arabic speakers also show greater difficulties spelling vowels, particularly short vowels (which are typically not represented in their L1 writing system) than other ESL students (Fender, 2008; Saigh and Schmitt, 2012). A similar pattern has also been found for L1 Hebrew speakers (Martin, 2017).

In sum, the limited research on ESL spelling abilities indicates that there is substantial overlap with L1 spelling development, including the reliance on phonological versus orthographic information. However, ESL spelling is also influenced by both L1 phonology (often leading to difficulties spelling non-native phonemes or distinguishing spellings for non-native phonemic contrasts) and the L1 writing system (e.g., reduced sensitivity to vowels in abjad L1 speakers; greater reliance on phonological skills in alphabetic L1 speakers and greater reliance on visual-orthographic skills in morphosyllabic L1 speakers).

## The Current Study

As detailed above, despite the centrality of spelling ability to overall literacy skill, relatively little research has examined ESL spelling abilities. Most of the research that has been conducted – like L1 spelling studies (Arndt and Foorman, 2010) – has focused on younger learners, not adults (Allaith and Joshi, 2011), and has not always included a comparison group of L1 English speakers. This makes the patterns of ESL spelling errors difficult to interpret (Fender, 2008; Saigh and Schmitt, 2012). In addition, this research has typically investigated only single L1 groups at a time (Figueredo, 2006), leading to difficulties in making accurate cross-linguistic comparisons of ESL spelling skills due to differences in task characteristics, target words, and lexical and learner characteristics (Ziegler and Goswami, 2005). Finally, little research has examined L2 spelling in relation to other key component literacy skills, such as phonological awareness, despite the fact that phonological awareness is a fundamental literacy skill for English and is closely connected with spelling ability in L1 English readers (Fender, 2008).

The current study was conducted in an effort to begin filling in these gaps. In contrast to much of the existing research, the target participants were adult learners of ESL at the university level (rather than children in elementary or middle school). Data were collected from learners with a range of L1 backgrounds; these were grouped into three main categories on the basis of L1 writing system: alphabet, abjad, or morphosyllabary. Collecting data from this range of L1 backgrounds facilitates cross-linguistic comparisons using uniform task items and procedures. In addition, data were collected from a comparison group of L1 English speakers, so that ESL performance (across L1 groups) could be understood in the context of native-level performance. Finally, participants also completed a phonological awareness task, so that spelling ability could be examined in relation to this key literacy skill. Phonological awareness was chosen because it is a critical component skill for literacy, particularly in the opaque orthography employed in English (e.g., Adams, 1990; Hanley et al., 2004; Ziegler and Goswami, 2005). It also has a reciprocal, mutually supportive relationship with spelling and reading ability (e.g., Read et al., 1986; Perfetti et al., 1987; Goswami and Bryant, 1990; Huang and Hanley, 1995; Burgess and Lonigan, 1998), and may provide insight regarding the strength of participants’ general phonological skills and the degree to which they rely on such skills for their L2 English spelling.

## Materials and Methods

### Participants

Data were collected from 70 participants: 47 ESL learners and 23 L1 English speakers (serving as a native speaker reference group). All participants were adult (18 years or older) students recruited from the intensive English program, undergraduate, and graduate student populations at a mid-sized regional university in the midwestern United States. The 47 ESL learners (23 female, 21 male, 3 with no data; average age 22.18 years) were either preparing for or enrolled in undergraduate or graduate studies. They were classified into three groups based on the type of writing system employed in their L1: 27 alphabetic L1 participants (24 L1 Spanish speakers, 1 L1 French speaker, 1 L1 Korean speaker, and 1 L1 Efik speaker); 7 abjad L1 participants (all L1 Arabic speakers); and 13 L1 morphosyllabary participants (all L1 Mandarin Chinese speakers). Specific age data were not available for the 23 L1 English speakers (19 female, 4 male), though all participants fell within the typical undergraduate student age range of 18–25 years old.

For the ESL learners, L2 proficiency information was available in the form of a score from one or both of two different standardized English proficiency tests: the Test of English as a Foreign Language (TOEFL) or the Oxford Online Placement Test (OOPT). Because there is no established, validated method of converting scores from one of these two assessments to an equivalent score on the other, the scores on each assessment were instead converted to the corresponding level of proficiency as described in the Common European Framework of Reference (Council of Europe, 2001): A1, A2, B1, B2, or C1 + (see Table 1 for detailed descriptions). Due to limited sample sizes for each L1 at each proficiency level, these CEFR levels were further combined into two main proficiency levels: lower proficiency (A1 through B1 levels) and higher proficiency (B2 through C1 + levels). This cut-off, of B1 vs. B2 levels as distinguishing higher versus lower proficiency, is consistent with the cut-offs used for undergraduate (B2) or graduate (C1) admissions at the institution where data were collected. There were 22 lower-proficiency and five higher-proficiency alphabetic L1 speakers, five lower-proficiency and two higher-proficiency abjad L1 speakers, and five lower-proficiency and eight higher-proficiency morphosyllabic L1 speakers.

**TABLE 1:** Common European Framework of References (CEFR) proficiency level descriptions.

*General description*	*Proficiency level*	*Description*
*Proficient User*	*C2*	*Can understand with ease virtually everything heard or read. Can summarize information from different spoken and written sources, reconstructing arguments and accounts in a coherent presentation. Can express him/herself spontaneously, very fluently and precisely, differentiating finer shades of meaning even in more complex situations.*
	*C1*	*Can understand a wide range of demanding, longer texts, and recognize implicit meaning. Can express him/herself fluently and spontaneously without much obvious searching for expressions. Can use language flexibly and effectively for social, academic and professional purposes. Can produce clear, well-structured, detailed text on complex subjects, showing controlled use of organizational patterns, connectors and cohesive devices.*
*Independent User*	*B2*	*Can understand the main ideas of complex text on both concrete and abstract topics, including technical discussions in his/her field of specialization. Can interact with a degree of fluency and spontaneity that makes regular interaction with native speakers quite possible without strain for either party. Can produce clear, detailed text on a wide range of subjects and explain a viewpoint on a topical issue giving the advantages and disadvantages of various options.*
	*B1*	*Can understand the main points of clear standard input on familiar matters regularly encountered in work, school, leisure, etc. Can deal with most situations likely to arise whilst traveling in an area where the language is spoken. Can produce simple connected text on topics which are familiar or of personal interest. Can describe experiences and events, dreams, hopes and ambitions and briefly give reasons and explanations for opinions and plans.*
*Basic User*	*A2*	*Can understand sentences and frequently used expressions related to areas of most immediate relevance (e.g., very basic personal and family information, shopping, local geography, employment). Can communicate in simple and routine tasks requiring a simple and direct exchange of information on familiar and routine matters. Can describe in simple terms aspects of his/her background, immediate environment and matters in areas of immediate need.*
	*A1*	*Can understand and use familiar everyday expressions and very basic phrases aimed at the satisfaction of needs of a concrete type. Can introduce him/herself and others and can ask and answer questions about personal details such as where he/she lives, people he/she knows and things he/she has. Can interact in a simple way provided the other person talks slowly and clearly and is prepared to help.*

*Descriptions are taken from the official site of the Council of Europe (https://www.coe.int/en/web/common-european-framework-reference-languages/table-1-cefr-3.3-common-reference-levels-global-scale).*

### Materials and Procedure

The first task completed by participants was a brief survey that elicited participants’ L1. Following this, participants completed two spelling dictation measures: one comprising 34 real English words and one comprising 16 English pseudowords (see the Supplementary Material for items and details of item characteristics). The 34 real word items were a subset of the items used by Fender (2008). They included 12 items targeting within-word spellings, 11 targeting syllable juncture spellings, and 11 targeting derivational spellings. The specific items chosen were selected to include a variety of orthographic features (e.g., consonant and vowel digraphs, silent < e >), syllable structures (e.g., single consonants and consonant clusters in onsets and rhymes), and number of syllables (2–4; average 2.12). Words had an average of 7.18 letters and 1.50 morphemes, and across all items there were 71 judgments of consistent syllables and 73 judgments of inconsistent syllables^1^. The 16 pseudoword items were taken from a variety of published studies examining pseudoword spelling in ESL/EFL learners (Liow and Poon, 1998; Wang and Geva, 2003b; San Francisco et al., 2006). These specific items were again chosen to represent a variety of orthographic features and syllable structures. Pseudowords were all monosyllabic and had an average of 4.31 letters and 3.50 phonemes. Across all items there were 14 judgments of consistent syllables and 18 judgments of inconsistent syllables.

Participants were tested in a group setting. Each participant was seated at an individual Macintosh computer in a computer lab. Participants completed the real word spelling dictation measure first. They were told that they would hear an English word pronounced aloud twice, with a pause in between, and given approximately 10–12 s to write down the word with the most accurate spelling they could. Following this, participants completed the pseudoword spelling dictation measure. For this task, they were told that they would hear a made-up word that was not a real English word, and that they should write down how they thought the word should be spelled if it were a real English word. Again participants heard each item pronounced aloud twice and were given approximately 10–12 s to write down the spelling. All items were pronounced carefully by a female native English speaker trained in ESL.

The final task completed by participants was a computer-adapted version of the Elision task from the Comprehensive Test of Phonological Processing (Wagner et al., 1999) to measure their phonological awareness. This task comprised six unscored practice items (with feedback), involving the deletion of a whole syllable from a disyllabic word (3), the deletion of the initial phoneme from a monosyllabic word (2), and the deletion of the final phoneme from a monosyllabic word (1). The initial unscored items were completed as a group, following the standard procedure describe in the Examiner’s Manual for the CTOPP. These were followed by 20 scored items (without feedback), completed individually by each student on their computer. The 20 scored items targeted the deletion of a variety of units: whole syllables from disyllabic words (3), single consonants in word-initial (3), medial (3), or final (2) positions, and consonants from within a consonant cluster in word initial (5), medial (2), or final (2) positions. Total testing time took approximately 30–60 min.

### Scoring and Analyses

Spelling responses were first typed verbatim into a digital spreadsheet for scoring and analysis. Any doubts about the written responses (e.g., due to unclear handwriting) were resolved through discussion between a trained research assistant and the first author. A strict scoring procedure (implemented via an Excel formula) was used to determine accuracy: if the participant’s production was spelled exactly correct it was marked as correct, and any deviations from the correct spelling resulted in an incorrect marking.

Responses to the Elision items were also transcribed into a digital spreadsheet for scoring and analysis. All responses to the Elision task were transcribed by a minimum of two trained research assistants; inter-rater reliability was initially 89% and items for which there was disagreement were resolved after consulting a third coder.

Pearson correlations and generalized linear mixed effects models with random intercepts for participants and items (implemented via the lme4 package in R, Bates et al., 2015) were used to analyze the data. A range of participant and item characteristics and the two-way interactions among them were initially considered for inclusion during model building: participants’ age and gender; spelling items’ length (in letters), consistency, and orthographic neighborhood; and real words’ frequency and mean bigram statistics. However, neither age nor gender had a significant relationship with any of the outcome variables (age: *r* = 0.17, *p* = 0.30 with word accuracy, *r* = 0.24, *p* = 0.15 with pseudoword accuracy, *r* = 0.21, *p* = 0.23 with elision accuracy; gender: *r* = 0.09, *p* = 0.47 with word accuracy, *r* = 0.18, *p* = 0.15 with pseudoword accuracy, *r* < 0.01, *p* = 0.98 with elision accuracy) and were thus not included in any of the mixed effects models.

Correlations were also used to guide the selection of item characteristics to consider in modeling word and pseudoword spelling accuracy. Although no item characteristics were significantly associated with pseudoword spelling accuracy (length in letters, *r* = −0.18, *p* = 0.50; number of orthographic neighbors, *r* = 0.34, *p* = 0.20; frequency of orthographic neighbors, *r* = −0.14, *p* = 0.61; positional biphone frequency, *r* = −0.05, *p* = 0.85, positional letter frequency, *r* = −0.06, *p* = 0.84; consistency, *r* = −0.17, *p* = 0.54), a number of item characteristics were significantly associated with real word spelling accuracy (length in letters, *r* = −0.52, *p* = 0.002; number of orthographic neighbors, *r* = 0.39, *p* = 0.02; number of phonological neighbors, *r* = 0.34, *p* = 0.049; age of acquisition, *r* = −0.52, *p* = 0.002). Thus, length and number of orthographic and phonological neighbors were considered in model building. Log frequency (*r* = 0.23, *p* = 0.20 with real word accuracy) was also considered due to its substantial influence in general lexical processing; however, age of acquisition was not included in models because of its high correlation with length (*r* = 0.81, *p* < 0.001) and concerns regarding issues with multicollinearity.

Finally, Pearson correlations were calculated to examine the relationships among real word spelling accuracy, pseudoword spelling accuracy, and phonological awareness skills; these correlations were calculated separately for each L1 background group in order to determine whether the different groups showed similar patterns of interrelatedness among these skills. For the ESL groups, two-step hierarchical regressions (blockwise entry with proficiency level dummy-coded as lower-proficiency vs. higher-proficiency entered first) were used to calculate semi-partial correlation coefficients between real word spelling accuracy, pseudoword spelling accuracy, and elision accuracy, controlling for L2 English proficiency.

## Results

### Accuracy

#### Relationship Between Spelling and Phonological Awareness Accuracy

Pearson correlations (zero-order for L1 English speakers, semi-partial for ESL groups) among real word spelling accuracy, pseudoword spelling accuracy, and elision accuracy are in Table 2. As can be seen, the patterns of interrelationships among these three tasks differed across all participant groups. The L1 English speakers had significant positive correlations between real word spelling accuracy, pseudoword spelling accuracy, and elision accuracy. Similar to the L1 English speakers, the alphabetic L1 speakers showed a significant correlation between real word spelling accuracy and phonological awareness. However, unlike the L1 English speakers, the alphabetic L1 speakers did not have significant correlations between pseudoword spelling accuracy and phonological awareness, or between real word and pseudoword spelling accuracy. The abjad L1 speakers had a different pattern: a significant correlation between pseudoword spelling accuracy and phonological awareness, but not between real word spelling accuracy and phonological awareness (though they also showed no significant correlation between real word and pseudoword spelling accuracy). The morphosyllabic L1 speakers, on the other hand, showed no significant correlations between any of these three tasks.

**TABLE 2:** Correlations among spelling and phonological awareness accuracy by L1 background.

L1 English (*n* = 23)		
	Real word accuracy	Pseudoword accuracy
Pseudoword accuracy	0.43*	−⁣−
Elision accuracy	0.50*	0.66**
**Alphabetic L1 (*n* = 27)**		
	Real word accuracy	Pseudoword accuracy
Pseudoword accuracy	0.17	−⁣−
Elision accuracy	0.36*	0.04
**Abjad L1 (*n* = 7)**		
	**Real word accuracy**	**Pseudoword accuracy**

Pseudoword accuracy	–0.29	−⁣−
Elision accuracy	–0.38	0.79*
**Morphosyllabic L1 (*n* = 13)**		
	**Real word accuracy**	**Pseudoword accuracy**

Pseudoword accuracy	–0.16	−⁣−
Elision accuracy	0.41	0.10

*p < 0.05; **p < 0.01. Correlations for the L1 English group are zero-order; correlations for the ESL groups are semi-partial (controlling for L2 English proficiency).

#### Phonological Awareness Accuracy

The means and standard deviations for Elision accuracy are given in Table 3. There was a significant overall effect of L1 writing system, χ^2^(*df* = 3) = 35.86, *p* < 0.001, and the L1 English speakers were significantly more accurate than all three ESL groups: alphabetic L1 speakers (β = −3.27, *z* = −6.50, *p* < 0.001), abjad L1 speakers (β = −2.11, *z* = 2.85, *p* = 0.004), and morphosyllabic L1 speakers (β = −1.45, *z* = −2.48, *p* = 0.01). Next, the ESL speaker groups were examined in more detail to explore the influence of both L1 writing system and L2 English proficiency on Elision accuracy. The overall effect of L1 writing system was significant, χ^2^(*df* = 2) = 8.11, *p* = 0.02. Specifically, the abjad L1 and morphosyllabic L1 speakers were both significantly more accurate than the alphabetic L1 speakers (β = 1.04, *z* = 2.07, *p* = 0.04; β = 1.08, *z* = 2.57, *p* = 0.01, respectively). However, the difference between the abjad L1 and morphosyllabic L1 speakers was not significant, β = 0.04, *z* = 0.07, *p* = 0.94. The effect of L2 English proficiency was also significant, β = 1.60, *z* = 3.97, *p* < 0.001, such that higher proficiency participants were significantly more accurate than lower proficiency participants. However, the interaction between L1 writing system and proficiency was not significant, χ^2^(*df* = 2) = 2.21, *p* = 0.33, suggesting that the effect of proficiency is the same regardless of L1 writing system.

**TABLE 3:** Mean word and pseudoword spelling accuracy and elision accuracy (% correct) by L1 background and ESL proficiency level.

	Real word spelling	Pseudoword spelling	Elision
L1 English	94.25 (7.18)	66.11 (15.32)	78.91 (23.50)
Alphabetic L1	59.63 (19.80)	12.96 (15.84)	36.49 (22.53)
Higher proficiency	84.71 (10.89)	32.50 (24.37)	63.33 (13.59)
Lower proficiency	53.94 (16.71)	8.52 (9.27)	30.39 (19.56)
Abjad L1	67.65 (21.94)	23.21 (10.02)	60.00 (20.82)
Higher proficiency	82.35 (12.48)	28.13 (13.26)	77.50 (31.82)
Lower proficiency	61.76 (23.07)	21.25 (9.48)	53.00 (13.51)
Morphosyllabic L1	76.85 (16.72)	18.10 (12.38)	64.62 (14.21)
Higher proficiency	84.93 (11.17)	22.37 (13.88)	70.63 (15.22)
Lower proficiency	63.92 (16.77)	11.25 (5.23)	55.00 (3.54)

*Standard deviations are given in parentheses.*

#### Spelling Accuracy

The means and standard deviations for real word and pseudoword spelling accuracy are given in Table 3. Overall spelling accuracy was first examined as a function of participant and item characteristics that were relevant for all participants and items: lexicality (real words vs. pseudowords), item length (in letters), orthographic neighborhood size; L1 writing system (alphabet, abjad, morphosyllabary, or L1 English), and elision accuracy. The effect of lexicality was significant, β = 3.18, *z* = 6.01, *p* < 0.001; the effect of length was marginally significant, β = −0.24, *z* = −1.80, *p* = 0.07; and the effect of orthographic neighborhood size was not significant, β = −0.004, *z* = −0.08, *p* = 0.93. The effect of elision accuracy was significant, β = 2.77, *z* = 5.71, *p* < 0.001, as was the overall effect of L1 writing system, χ^2^(*df* = 12) = 69.51, *p* < 0.001. Critically, L1 writing system also interacted significantly with lexicality, χ^2^(*df* = 3) = 13.50, *p* = 0.004. Therefore, further analyses were conducted for real words and pseudowords separately.

For real word spelling, model building initially considered all participant characteristics (L1 writing system, elision accuracy) and item characteristics (length, number of orthographic neighbors, and phonological neighbors) that correlations had suggested may be relevant, as well as log frequency. The overall effect of L1 writing system was significant, χ^2^(*df* = 3) = 25.11, *p* < 0.001, as were the effects of elision accuracy (β = 3.19, *z* = 4.63, *p* < 0.001) and word length (β = −0.51, *z* = −2.81, *p* = 0.01). In addition, there were two significant interactions involving comparisons between the L1 English speakers and the alphabetic L1 speakers (by orthographic neighborhood size, β = −0.29, *z* = −2.47, *p* = 0.01, and by phonological neighborhood size, β = −0.15, *z* = −2.46, *p* = 0.01) as well as two marginally significant interactions involving comparisons between the L1 English speakers and the abjad L1 speakers (by orthographic neighborhood size, β = −0.26, *z* = −1.89, *p* = 0.06, and by log frequency, β = 0.38, *z* = 1.72, *p* = 0.08). Thus, the final models of real word spelling accuracy examined L1 English speakers and the ESL speakers separately, and L2 English proficiency was also added as a factor for the ESL model.

These final models are given in Table 4; these models were selected as the final models because they each had significantly better model fit (determined using the log-likelihood ratio test and AIC values) than alternative models that included non-significant predictors. For L1 English speakers, there were only two significant predictors: elision accuracy and item length. Specifically, participants with higher phonological awareness scores had higher spelling accuracy, and longer words were spelled less accurately than shorter words. For the ESL speakers, several factors significantly impacted real word spelling accuracy. The overall effect of L1 writing system was not significant, χ^2^(*df* = 2) = 0.20, *p* = 0.91 (though it was maintained in the model due to significant interactions with other variables). There were significant effects of proficiency and elision accuracy, such that participants with higher L2 English proficiency and with higher phonological awareness had significantly higher spelling accuracy than participants with lower L2 English proficiency and with lower phonological awareness. There were also significant effects of orthographic and phonological neighborhood size, with lower spelling accuracy for words with larger orthographic or phonological neighborhoods.

**TABLE 4:** Final linear mixed effects models predicting real word spelling accuracy in L1 English speakers and ESL speakers.

	L1 English speakers			ESL speakers		
	β	*SE*	*z*	β	*SE*	*z*
Intercept	6.19	1.88	3.30***	2.31	1.65	1.40
**Fixed effects**						
Abjad L1^a^				2.75	1.16	2.38*
Morphosyllabic L1^a^				1.39	1.39	1.00
Higher proficiency^b^				1.29	0.39	3.40***
Elision accuracy	2.77	1.17	2.36*	2.51	0.97	2.58*
Length	–0.53	0.20	−2.71**	–0.12	0.28	–0.43
Orthographic neighborhood				–1.00	0.40	−2.52*
Phonological neighborhood				–0.62	0.24	−2.57*
Log frequency				0.13	0.20	0.67
Abjad L1^a^*Elision accuracy				–4.82	1.93	−2.50*
Morphosyllabic L1^a^*Elision accuracy				2.24	2.29	0.98
Orthographic neighborhood* Elision accuracy				0.35	0.16	2.21*
Phonological neighborhood* Elision accuracy				0.19	0.07	2.64**
Orthographic neighborhood*Length				0.14	0.08	1.78^‡^
Phonological neighborhood*Length				0.09	0.05	1.99*
Orthographic neighborhood*Log frequency				–0.36	0.15	−2.41*
Phonological neighborhood*Log frequency				–0.18	0.08	−2.40*

	**Variance Component**		***SD***	**Variance Component**		***SD***
Random effects						

Participants Intercept	0.99		1.00	0.57		0.76
Items Intercept	2.53		1.59	0.79		0.89

^*a*^Alphabetic L1 is the reference group. ^*b*^Lower proficiency is the reference group. ^‡^*p* < 0.10; *p < 0.05; **p < 0.01, ***p < 0.001.

There were also a number of significant interactions that qualified these overall effects. The interaction between L1 writing system and elision accuracy was significant, χ^2^(*df* = 2) = 7.82, *p* = 0.02; follow-up analyses within each L1 writing system group were used to examine this further. For the alphabetic and morphosyllabic L1 participants, the effect of phonological awareness was significant and positive: participants with higher elision accuracy had higher real word spelling accuracy (alphabetic L1: β = 2.60, *z* = 2.28, *p* = 0.02; morphosyllabic L1: β = 7.91, *z* = 2.63, *p* = 0.01). In contrast, for the abjad L1 participants, the effect of phonological awareness was marginally significant, but in the opposite direction: abjad speakers with higher elision accuracy had *lower* real word spelling accuracy, β = −4.60, *z* = −1.89, *p* = 0.06.

The interactions between elision accuracy and orthographic neighborhood size and phonological neighborhood size were also significant. In each case, participants who had higher phonological awareness experienced a larger effect of both orthographic neighborhood size and phonological neighborhood size. Finally, the interactions between word frequency and orthographic neighborhood size and phonological neighborhood size were significant. These interactions indicate that higher-frequency words were associated with smaller effects of both orthographic neighborhood size and phonological neighborhood size.

For pseudoword spelling, model building considered the same participant characteristics (L1 writing system, elision accuracy) and applicable item characteristics (length, number of orthographic neighbors) that were considered with real word accuracy so that the results could be directly compared. The overall effect of L1 writing system was significant, χ^2^(*df* = 3) = 50.97, *p* < 0.001, as was the main effect of elision accuracy, β = 2.22, *z* = 4.36, *p* < 0.001. In addition, there were two significant interactions involving comparisons between the L1 English speakers and the abjad L1 speakers (by length, β = 3.24, *z* = 3.45, *p* < 0.001, and by orthographic neighborhood size, β = −0.21, *z* = −2.41, *p* = 0.02) as well as two significant interactions involving comparisons between the L1 English speakers and the morphosyllabic L1 speakers (again by length, β = 1.97, *z* = 2.94, *p* = 0.003, and by orthographic neighborhood size, β = −0.23, *z* = −3.67, *p* < 0.001). Thus, the final models of pseudoword spelling accuracy again considered L1 English speakers and the ESL speakers separately, and L2 English proficiency was added as a factor for the ESL model.

These final models are given in Table 5; these models were again selected as the final models because they each had significantly better model fit (determined using the log-likelihood ratio test and AIC values) than alternative models that included non-significant predictors. For L1 English speakers, there was only one significant predictor: elision accuracy. As with real word spelling, participants with higher phonological awareness had higher pseudoword spelling accuracy. Also similar to the results for real word spelling, there were several factors that significantly impacted pseudoword spelling accuracy for the ESL speakers. The overall effect of L1 writing system was not significant, χ^2^(*df* = 2) = 2.91, *p* = 0.23 (though it was again maintained in the model due to significant interactions with other variables). There was also a significant effect of proficiency, such that participants with higher L2 English proficiency had significantly higher pseudoword spelling accuracy than participants with lower L2 English proficiency.

**TABLE 5:** Final linear mixed effects models predicting pseudoword spelling accuracy in L1 English speakers and ESL speakers.

	L1 English speakers			ESL speakers		
	β	*SE*	*z*	β	*SE*	*z*
Intercept	–0.81	0.43	−1.88^‡^	–2.39	2.90	–0.83
**Fixed effects**						
Abjad L1^a^				12.73	4.76	2.67**
Morphosyllabic L1^a^				7.23	3.49	−2.07*
Higher proficiency^b^				1.00	0.42	2.39*
Elision accuracy	1.99	0.50	3.95***	1.11	1.04	1.07
Length				0.12	0.65	0.85
Orthographic neighborhood				–0.05	0.07	–0.73
Abjad L1^a^*Length				2.97	1.06	2.82**
Morphosyllabic L1^a^*Length				1.53	0.78	1.96*
Abjad L1^a^*Orthographic Neighborhood				–0.17	0.10	−1.81^‡^
Morphosyllabic L1^a^*Orthographic Neighborhood				–0.20	0.07	−2.67**

	**Variance Component**		***SD***	**Variance Component**		***SD***

**Random effects**						
Participants Intercept	0.004		0.06	0.41		0.64
Items Intercept	0.40		0.64	0.77		0.88

^*a*^Alphabetic L1 is the reference group. ^*b*^Lower proficiency is the reference group. ^‡^*p* < 0.10; *p < 0.05; **p < 0.01, ***p < 0.001.

There were two significant interactions that qualified these overall effects: between L1 writing system and length, χ^2^(*df* = 2) = 12.09, *p* = 0.002; and between L1 writing system and orthographic neighborhood size, χ^2^(*df* = 2) = 9.58, *p* = 0.01. Follow-up analyses within each L1 writing system group were used to examine these interactions further. For the alphabetic L1 participants, neither the effect of length (β = 0.002, *z* = 0.002, *p* > 0.99) nor the effect of orthographic neighborhood size (β = −0.05, *z* = −0.65, *p* = 0.52) were significant. However, within this alphabetic L1 model, the effect of proficiency was significant, β = 1.50, *z* = 1.98, *p* = 0.048, with higher pseudoword spelling accuracy among higher proficiency participants. For the abjad L1 participants, the effect of length was significant (β = 2.77, *z* = 2.33, *p* = 0.02), with longer pseudowords being spelled more accurately than shorter pseudowords. The effect of orthographic neighborhood size was marginally significant (β = −0.18, *z* = −1.71, *p* = 0.09), with larger orthographic neighborhoods associated with somewhat lower pseudoword spelling accuracy, but the effect of proficiency was not significant and was dropped from the final abjad L1 model. Finally, for the morphosyllabic L1 participants, the effect of length was significant (β = 1.65, *z* = 2.03, *p* = 0.04), again with longer pseudowords being spelled more accurately than shorter pseudowords. The effect of orthographic neighborhood size was also significant (β = −0.24, *z* = −3.02, *p* = 0.002), again with larger orthographic neighborhoods associated with lower pseudoword spelling accuracy, though the effect of proficiency was not significant and also dropped from the final morphosyllabic L1 model.

### Error Analyses

Following the quantitative analysis of overall (strict) accuracy described above, a more in-depth investigation into the *types* and *quantities* of spelling errors committed by participants was conducted. First, the spelling of each grapheme in each item was examined to determine whether it was correct or incorrect. Generally following Masterson and Apel (2010), graphemes were defined as the letter or sequence of letters representing each spoken phoneme in the target items. For example, *dress* was divided into the following graphemes corresponding to phonemes: d| r| e| ss; *separate* was divided into s| e| p| a| r| aCe| t (with aCe representing the non-linear sequence of vowel + consonant + < e > indicating long vowel); and *knowledge* was divided into kn| ow| l| e| dge.

Incorrect spellings of graphemes were further categorized in terms of whether the particular sound was misspelled or missing or whether additional sounds were represented in the spelling that were not present in the dictated word. More specifically, the categories were as follows (with examples drawn directly from the data): incorrect consonant grapheme (the wrong letter(s) representing a consonant phoneme in the word, e.g., < napcen > for *napkin*); incorrect vowel grapheme (the wrong letter(s) representing a vowel phoneme in the word, e.g., < seperate > for *separate*); missing consonant grapheme (no letter(s) present to represent a consonant phoneme in the word, e.g., < resonsible > for *responsible*); missing vowel grapheme (no letter(s) present to represent a vowel phoneme in the word, e.g., < decsion > for *decision*); extra consonant grapheme (additional letter(s) representing a consonant phoneme not present in the word, e.g., < grownd > for *grown*); or extra vowel grapheme (additional letter(s) representing a vowel phoneme not present in the word, e.g., < recoganize > for *recognize*). Inter-rater reliability was initially 91% for real words and 63% for pseudowords; items for which there was disagreement were resolved through discussion among coders.

Similar to the main analyses examining overall accuracy, linear mixed effects analyses were used, this time to examine the *rate of errors per item* (rather than strict accuracy) as a function of L1 background, L2 English proficiency, and phonological awareness. The average number of errors per item of different types are given in Table 6; errors per item was used as the dependent variable in order to make error rates comparable between real words and pseudowords, given the different number of each type of item (34 real words versus 16 pseudowords).

**TABLE 6:** Error analyses - mean error rates (per item) to different item types (real words, pseudowords) and segment types (vowels, consonants) by L1 background and ESL proficiency level.

	Real words	Pseudowords	Vowels	Consonants
L1 English	0.07 (0.09)	0.43 (0.22)	0.12 (0.12)	0.10 (0.11)
Alphabetic L1	0.59 (0.37)	0.87 (0.31)	0.23 (0.18)	0.24 (0.19)
Higher proficiency	0.21 (0.21)	0.93 (0.33)	0.28 (0.25)	0.16 (0.16)
Lower proficiency	0.68 (0.35)	0.86 (0.31)	0.22 (0.16)	0.26 (0.20)
Abjad L1	0.66 (0.68)	1.06 (0.23)	0.45 (0.28)	0.29 (0.22)
Higher proficiency	0.30 (0.10)	0.94(<0.001)	0.32 (0.18)	0.18 (0.11)
Lower proficiency	0.81 (0.77)	1.11 (0.26)	0.51 (0.30)	0.33 (0.24)
Morphosyllabic L1	0.41 (0.40)	1.12 (0.28)	0.38 (0.27)	0.23 (0.20)
Higher proficiency	0.25 (0.28)	1.03 (0.30)	0.32 (0.25)	0.20 (0.18)
Lower proficiency	0.66 (0.46)	1.24 (0.21)	0.46 (0.28)	0.29 (0.23)

*Standard deviations are given in parentheses. Error rates per item were calculated by summing the total number of errors to real words or to pseudowords, and the total number of errors involving vowels or involving consonants, and dividing this total by the number of items of that type (total number of real words, total number of pseudowords, or total number of items overall).*

#### Errors to Real Words Versus Pseudowords

The first error analysis examined the errors made to real words versus pseudowords (lexicality). The effect of lexicality was significant β = −0.36, *t* = 4.40, *p* < 0.001, with significantly fewer errors made to real words than pseudowords. The overall effect of L1 writing system was also significant, χ^2^(*df* = 3) = 34.50, *p* < 0.001, as was the effect of phonological awareness, β = −0.44, *t* = −3.17, *p* = 0.002, with higher phonological awareness associated with fewer errors. The interaction between L1 writing system and item type was also significant, χ^2^(*df* = 3) = 9.66, *p* = 0.02. Thus, L1 English speakers and ESL speakers were further examined separately, with L2 English proficiency also added as a factor for the ESL model.

For the L1 English speakers, the effect of phonological awareness was significant, β = −54, *t* = −0.19, *p* < 0.001, with higher phonological awareness associated with fewer errors. The effect of lexicality was also significant, with fewer errors made to real words than pseudowords, β = −0.61, *t* = 4.07, *p* < 0.001. The interaction between phonological awareness and lexicality was only marginally significant, β = 0.31, *t* = 1.70, *p* = 0.096; the trend was such that the effect of phonological awareness was slightly stronger for real words than it was for pseudowords.

For the ESL speakers, the overall effect of L1 writing system was not significant, χ^2^(*df* = 2) = 3.28, *p* = 0.19. In addition, the effect of proficiency was not significant, β = −0.03, *t* = −0.30, *p* = 0.77. However, the effect of lexicality was significant, β = −0.74, *t* = −6.59, *p* < 0.001, with fewer errors made to real words than to pseudowords. The interaction between lexicality and proficiency was also significant, β = 0.49, *t* = 3.55, *p* < 0.001, with more errors made to real words than pseudowords among lower proficiency speakers, but more errors made to pseudowords than to real words among higher proficiency speakers.

#### Errors Involving Consonants Versus Vowels

For the second error analysis, the incorrect, missing, and additional consonant errors and vowel errors were summed together by phoneme type to calculate the total number of consonant-related errors and vowel-related errors for each participant. These were then examined across L1 groups and L2 English proficiency to determine whether there were any differential accuracies by segment type (consonant versus vowel sounds), as has been demonstrated in previous research with abjad L1 speakers (e.g., Fender, 2008; Saigh and Schmitt, 2012; Martin, 2017).

There was a significant overall effect of L1 writing system, χ^2^(*df* = 3) = 27.58, *p* < 0.001. The main effect of lexicality was not significant, β = 0.10, *t* = 1.42, *p* = 0.16, though the main effect of segment type was marginally significant, β = 0.05, *t* = 1.66, *p* = 0.099, with a slight trend toward more vowel errors than consonant errors. However, these main effects were qualified by a number of significant interactions, including between L1 writing system and lexicality, χ^2^(*df* = 3) = 18.38, *p* < 0.001 and between L1 writing system and segment type, χ^2^(*df* = 3) = 15.44, *p* = 0.001. Due to these significant interactions with L1 writing system, as with previous analyses, the L1 English speakers and ESL speakers were examined in more detail separately.

For the L1 English speakers, only phonological awareness and lexicality were significant predictors: phonological awareness, β = −0.12, *t* = −3.08, *p* = 0.01; lexicality, β = −0.15, *t* = −9.30, *p* < 0.001. Specifically, higher phonological awareness and real words were associated with fewer errors than were lower phonological awareness and pseudowords. Notably, the effect of segment type was not significant.

For the ESL speakers, the overall effect of L1 writing system was again significant, χ^2^(*df* = 2) = 7.26, *p* = 0.03. However, a number of main effects were not significant: proficiency, β = −0.02, *t* = −0.33, *p* = 0.75; phonological awareness, β = 0.07, *t* = 0.52, *p* = 0.60; lexicality, β = 0.01, *t* = 0.10, *p* = 0.92; and segment type, β = 0.04, *t* = 1.11, *p* = 0.27. Importantly, these main effects were qualified by a significant interaction between L1 writing system and segment type, χ^2^(*df* = 2) = 14.44, *p* < 0.001. Thus, each L1 writing system group was examined separately.

For alphabetic L1 speakers, most main effects were not significant: proficiency, β = −0.05, *t* = −0.53, *p* = 0.60; phonological awareness, β = 0.04, *t* = 0.23, *p* = 0.82; and lexicality, β = −0.04, *t* = −0.40, *p* = 0.69. The main effect of segment type was marginally significant, β = 0.11, *t* = 1.97, *p* = 0.05, with somewhat more errors made involving vowels than consonants. However, there were a number of significant interactions among these variables. Proficiency interacted with lexicality, β = 0.20, *t* = 2.63, *p* = 0.01, such that there was no lexicality effect in higher proficiency participants, but there were more errors per item to real words than to pseudowords in lower proficiency participants. Proficiency also interacted with segment type, β = −0.15, *t* = −2.39, *p* = 0.02, such that higher proficiency participants made somewhat more errors involving vowels than consonants, but there was essentially no difference in lower proficiency participants. Lastly, phonological awareness interacted with lexicality, β = −0.35, *t* = −2.55, *p* = 0.01, such that phonological awareness was not related to the error rate among pseudowords, but higher phonological awareness was associated with a lower error rate among real words.

For abjad L1 speakers, the effect of lexicality was marginally significant, β = −0.13, *t* = −1.88, *p* = 0.08, with a lower error rate among real words than pseudowords. The effects of proficiency and phonological awareness were not significant and thus removed from the final model. However, the effect of segment type was significant, β = 0.16, *t* = 2.45, *p* = 0.02: abjad L1 speakers made significantly more errors involving vowels than consonants.

For morphosyllabic L1 speakers, the effect of lexicality was significant, β = −0.17, *t* = −2.66, *p* = 0.01, with a lower error rate among real words than pseudowords. The main effect of segment type was also significant, β = 0.24, *t* = 3.72, *p* < 0.001, with more errors involving vowels than consonants. As with the abjad L1 speakers, the effects of proficiency and phonological awareness were not significant and thus removed from the final model. However, the interaction between lexicality and segment type was also significant, β = −0.18, *t* = −2.05, *p* = 0.047; there were substantially more errors involving vowels than consonants among pseudoword items, but this difference was minimal among real words.

### Summary of Results

The correlations among real word spelling accuracy, pseudoword spelling accuracy, and phonological awareness accuracy showed different patterns in each L1 group. For the L1 English speakers, all three tasks were significantly positively correlated with one another, whereas for the morphosyllabic L1 speakers, there were no significant correlations among these three tasks. The alphabetic and abjad L1 speakers fell somewhere in the middle: phonological awareness accuracy was significantly correlated with real word spelling accuracy for the alphabetic L1 speakers and was significantly correlated with pseudoword spelling accuracy for the abjad L1 speakers, though these were the only significant correlations in these groups.

Regarding spelling accuracy, the native speaker comparison group (L1 English speakers) had substantially higher real word and pseudoword spelling accuracy than the three ESL groups. Interestingly, the L1 English speakers had relatively few predictors of spelling accuracy: only phonological awareness (for both real words and pseudowords) and length (real words only). No other lexical characteristics influenced L1 English speaker spelling accuracy.

The picture was much more complex among the ESL participants. L2 English proficiency was consistently related to both real word and pseudoword spelling accuracy, such that spelling accuracy increased along with increasing L2 proficiency. Similarly, higher phonological awareness scores were associated with higher real word spelling accuracy in the alphabetic and morphosyllabic L1 speakers, but the opposite pattern was found for the abjad L1 speakers: higher phonological awareness was associated with lower real word spelling accuracy. In contrast, higher phonological awareness scores were associated with higher pseudoword spelling accuracy across L1 groups. Lexical characteristics also influenced ESL spelling accuracy: higher phonological awareness scores were associated with larger effects of orthographic and phonological neighborhood size in real words, whereas higher word frequency was associated with smaller effects of orthographic and phonological neighborhood size in real words. Among pseudowords, the abjad and morphosyllabic L1 speakers had more accurate pseudoword spelling for *longer* pseudowords and for those with smaller orthographic neighborhoods.

Considering the phonological awareness scores themselves, the L1 English speakers had higher accuracy than all ESL groups on the elision task. Among the ESL participants phonological awareness accuracy increased along with higher L2 English proficiency across L1s. However, the alphabetic L1 speakers were significantly less accurate in their elision performance than the abjad and morphosyllabic L1 speakers, who did not differ from one another.

Finally, the L1 English speakers and ESL speakers also showed different patterns regarding the rates at which they made specific types of spelling errors. Among L1 English speakers, the only two patterns that emerged were that higher phonological awareness was related to a significantly lower error rate, and that the error rate was lower to real words than pseudowords. Once again, the pattern of results was more complex when considering the ESL speakers. Considering error rates to real words versus pseudowords, there was no overall effect of L1 writing system or L2 English proficiency level, but there was an unexpected interaction between lexicality and proficiency, such that lower proficiency ESL speakers had a higher error rate to real words than pseudowords, whereas higher proficiency ESL speakers had a higher error rate to pseudowords than real words. Considering error rates involving vowels versus consonants, there was a general tendency for a higher error rate to vowels than consonants. This was found among higher proficiency alphabetic L1 speakers, with pseudowords in morphosyllabic L1 speakers, and in abjad L1 speakers regardless of proficiency level or item type.

## Discussion

The current study examined real word and pseudoword spelling accuracy in adult learners of ESL from a variety of L1 backgrounds. To facilitate cross-linguistic comparisons, these ESL speakers were grouped by L1 writing system: alphabetic L1s, abjad L1s, and morphosyllabic L1s. Participants also completed the Elision subtest from the CTOPP to measure their phonological awareness. Analyses focused on the relationships among real word and pseudoword spelling and phonological awareness skills, detailed analyses of the types of errors made by participants, and the variations in these patterns across L1 groups.

As a whole, the results were largely consistent with the findings from previous research on ESL spelling abilities. The comparison group of L1 English speakers consistently had higher spelling accuracy (for both real words and pseudowords), fewer errors per item, and higher phonological awareness accuracy than all three ESL groups. Considering the three ESL groups, previous research has generally found that ESL speakers from a morphosyllabic L1 typically have relatively strong real word spelling skills, particularly for irregular/exception words, but have substantial difficulties reading and spelling pseudowords, whereas ESL speakers from an alphabetic L1 typically have stronger phonological skills and rely on them for word reading and spelling, resulting in a substantial number of overregularization errors (e.g., Wang and Geva, 2003b; Wang et al., 2003; Wang and Koda, 2007; Hamada and Koda, 2008; Leong, 2011). The results from the current study are partially consistent with these previous findings. Among the ESL groups, the morphosyllabic L1 group did have the highest real word spelling accuracy (77%), and very low pseudoword spelling accuracy (18%). In comparison, the alphabetic L1 speakers had the lowest real word spelling ability (60%), though they also had the lowest pseudoword spelling accuracy (13%) and phonological awareness accuracy (36%).

Given the relatively strong phonological skills that are typically found in learners with an alphabetic L1, it may initially seem surprising that the alphabetic L1 speakers in this study had such low pseudoword spelling and phonological awareness performance. However, there are a number of factors that can help explain this result. First, as discussed further below, the distribution of participants with different L1 writing systems was not even across proficiency levels, and there were more alphabetic L1 speakers at the lowest levels of proficiency (A1, A2) than in the other ESL groups. Given that spelling accuracy was also significantly related to L2 English proficiency, it is likely that the overall lower proficiency level of the alphabetic L1 speakers contributed to their lower raw scores. This interpretation is supported by the fact that, when L2 English proficiency was considered, the differences among the ESL groups were much smaller and real word spelling accuracy did not differ between the alphabetic and morphosyllabic L1 speakers.

Another contributing factor is likely the nature of phonological skill development in L1 Spanish (by far the largest L1 group among the alphabetic L1 speakers). Due in part to the highly consistent nature of the Spanish orthography, fine-grained phonological awareness skills (e.g., below the syllable level) are not needed for Spanish literacy and thus may not develop readily (e.g., Anthony et al., 2003, 2011). In addition, previous research has shown that readers of a consistent L1 orthography, such as Spanish, may transfer this expectation of consistency to their L2 (Haggan, 1993; Sun-Alperin and Wang, 2008; see also Branum-Martin et al., 2012). This expectation can hamper such individuals’ ability to adjust to the highly inconsistent nature of English, resulting in lower word recognition and spelling skills than may otherwise be expected – as was found in the current study.

It must also be noted that, although the alphabetic L1 speakers had low strict spelling accuracy, a different picture emerges if error rates per item are considered. In word spelling, the alphabetic L1 speakers frequently had low overall rates of errors per word, particularly on pseudowords. Thus, although their spellings may not have been accurate in terms of strict coding, the spellings produced by the alphabetic L1 speakers were relatively more target-like than those produced by the other ESL groups. This finding also highlights another important point: the conclusion that is reached from research results may be heavily dependent on the format of the task (e.g., Anthony et al., 2003, 2011), the types of items included (e.g., McBride-Chang, 1995; Juffs and Martin, 2014), and the way that responses are scored (e.g., Masterson and Apel, 2010; Clemens et al., 2014). Given the increasing recognition that the same tasks and items that have been developed for L1 research are not necessarily valid for ESL users and adult readers (Greenberg et al., 1997, 2002, 2009; Grant et al., 2012; Pae et al., 2012; Nanda et al., 2014), directly examining the influence of different scoring approaches will be critical for L2 literacy research going forward.

Examination of the results for error rates also highlights another initially unexpected pattern in the findings: a higher error rate to real words than to pseudowords, particularly in lower proficiency learners. Given that real words may be known, but pseudowords by definition are unknown, it might be expected that spellings, regardless of scoring approach, would be more accurate for real words than pseudowords. However, a closer examination of the characteristics of English words and pseudowords themselves helps illustrate why this may not always be the case. English spelling is notoriously opaque (Ziegler and Goswami, 2005; Share, 2008; Frost, 2012), meaning that real words naturally have wide variability in their consistency (see also Ziegler et al., 1997), and multimorphemic words in particular may have spellings that differ noticeably from expectations based on phoneme-grapheme correspondences alone (Helman et al., 2012; Bear et al., 2019). On the other hand, pseudowords may vary in the consistency of their component graphemes, but by the nature of their being pronounceable pseudowords, follow patterns that are already attested elsewhere in English. Thus, real words – including the multisyllabic and derived words used in the current study – may actually appear *less* consistent than pseudowords. If these items are not well-known words for participants (as may be the case for those with lower levels of proficiency), they are likely to be more difficult to spell and to result in an increased error rate. However, with increased proficiency (and increased vocabulary knowledge), this effect should diminish or disappear – as is attested in the current data.

The differential relationships between spelling and phonological skills found in the current study are also consistent with previous research. In the L1 English speakers, both real word and pseudoword spelling accuracy were significantly related to phonological awareness skills. This is consistent with the continuing importance of phonological skills for literacy in the opaque English orthography, compared to other languages in which the importance of phonological awareness decreases rapidly with increasing reading proficiency (e.g., Ziegler and Goswami, 2005). Consistent with previous findings that alphabetic L1 speakers rely relatively more on phonological skills for spelling and that non-alphabetic L1 speakers rely on phonological skills relatively less (e.g., Holm and Dodd, 1996; Wade-Woolley, 1999; McBride-Chang et al., 2004; Mayer et al., 2007), the alphabetic L1 speakers from this study showed a significant relationship between real word spelling accuracy and phonological awareness. In contrast, the abjad L1 speakers did not show a significant association between real word spelling accuracy and phonological awareness, and the morphosyllabic L1 speakers did not show any significant correlations between spelling accuracy and phonological awareness.

Examining the varying relationships between spelling and phonological skills is also an area where additional research with a larger number of participants at both lower and higher proficiency levels would be beneficial. In the current results there was an unexpected finding of a *negative* beta weight for elision accuracy as a predictor of real word spelling accuracy among the abjad L1 speakers, in contrast to the positive beta weights for the alphabetic and morphosyllabic L1 speakers. Based on a reviewer recommendation, we examined whether proficiency was a significant predictor of elision accuracy, and whether this relationship was different among the ESL groups. This is in fact what we found: there was a significant positive relationship between proficiency and elision accuracy for the alphabetic L1 (β = 2.22, *z* = 3.32, *p* = 0.001) and morphosyllabic L1 speakers (β = 1.20, *z* = 2.28, *p* = 0.02) but not for the abjad L1 speakers (β = 0.87, *z* = 0.91, *p* = 0.37). Thus, larger and more detailed datasets would be beneficial in future research to more directly explore these interactions and what they can tell us about L1 differences in literacy skills.

The results from the current study do provide new data to contribute to an unresolved issue in L2 spelling research: whether (L1-specific) errors decrease with increasing L2 proficiency, or whether they persist over continued L2 development. Some previous studies have found that such errors do decrease with increasing L2 proficiency (e.g., Fashola et al., 1996; Wang and Geva, 2003a), whereas others have found that spelling errors persist across grades (e.g., Zutell and Allen, 1988; Allaith and Joshi, 2011). The results from this study indicated a strong relationship between L2 English proficiency and spelling accuracy, measured via both strict accuracy and error rates per item. Further, after controlling for proficiency, L1 writing system differences persisted, considering both real word and pseudoword strict spelling accuracy and error rates. Thus, this study provides evidence for the continued influence of L1 literacy experiences on L2 (English) literacy skills across the development of L2 (English) proficiency.

Another question addressed by the current study was whether there would be significant L1 differences in accuracy spelling consonant versus vowel phonemes, as has been found for both L1 Arabic and L1 Hebrew speakers in prior research (e.g., Fender, 2008; Saigh and Schmitt, 2012; Martin, 2017). Broadly, this finding was confirmed: the error rate involving vowels was significantly higher than the error rate involving consonants, and this pattern was most widespread among the abjad L1 speakers. The alphabetic L1 speakers showed this pattern, but only among higher proficiency learners, whereas the morphosyllabic L1 speakers showed this pattern, but only with pseudowords. In contrast, the abjad L1 speakers showed a significantly higher error rate involving vowels, regardless of proficiency level or item type. Thus, the results of the current study confirm this pattern from previous research, and also show that other L1 groups may experience similar difficulties in at least some conditions (see also Martin, 2017).

Although this study makes important contributions toward our understanding of adult ESL spelling abilities, there are still a number of limitations that must be acknowledged. First, although data were collected from a wide range of L2 English proficiency levels and three representative L1 writing systems, there were relatively small sample sizes in some combinations of L2 English proficiency and L1 writing system. In addition, the number of participants from each L1 writing system was not consistent across all levels of proficiency, with somewhat more alphabetic L1 speakers at lower levels of proficiency compared to the abjad L1 and morphosyllabic L1 groups. Future research would benefit from a larger sample size, including targeted recruitment of a balanced sample of participants across L1 writing systems and proficiency levels; this was unfortunately not possible given the current context of data collection. Such research will be useful for confirming the specific findings from this study.

Another improvement that could be made by future research would be to include a wider variety of target items, both real words and pseudowords, and to specifically consider lexical characteristics during item selection. The items chosen for the current study were based on items previously used successfully in published research on ESL spelling abilities, but were not chosen with regard to their frequency, orthographic neighborhood size, or phonological neighborhood size. However, modeling the results for spelling accuracy suggested that these three lexical characteristics in particular may influence ESL spelling abilities, and are thus deserving of dedicated attention in future research.

A strength of the current study was the use of a dictation task to assess spelling ability. Although this type of assessment is common in L1- and child-focused literacy research, it is much lesson common in adult L2 research, and using it in this study eliminated the influence of learner avoidance found in studies of spelling errors from naturalistic writing samples (Allaith and Joshi, 2011). However, the words were not selected to target specific (or representative) phonemes, as has been done in some previous research (e.g., Wang and Geva, 2003a, b; Allaith and Joshi, 2011; Saigh and Schmitt, 2012). Thus, the particular difficulties that learners had may have been somewhat an artifact of the particular items that were included, and thus not fully representative of overall spelling abilities. A final limitation that we would like to highlight is the challenge of using standardized assessments, such as the Elision task from the CTOPP, with L2 speaker populations (see also Greenberg et al., 2009; Pae et al., 2012; Nanda et al., 2014; Winke et al., 2018). For example, when coding spoken responses to such assessments, it can be challenging to determine accuracy while accounting for L2 accents in a consistent – and fair – way. As the substantial difference between L1 alphabetic speakers’ spelling performance – measured via strict spelling accuracy versus error rates per item – demonstrates, issues of test validity and scoring reliability need to be more widely examined in L2 research.

## Conclusion

Although spelling is a critical literacy skill it has received relatively little attention, especially in the domain of cross-linguistic L2 literacy research. The current study sought to address a number of gaps left by existing research, by focusing on adult ESL users (rather than children), considering not only real word spelling ability but also pseudoword spelling ability and phonological awareness, and using the same materials and procedure with participants from a variety of L1 backgrounds (thus facilitating cross-linguistic comparisons). In many ways the results were consistent with previous research, demonstrating relatively high (real word) spelling accuracy yet somewhat weak phonological skills in morphosyllabic L1 speakers; more reliance on phonological skills in alphabetic L1 speakers; and significantly more errors involving vowels than consonants in abjad L1 speakers. However, there were also some unexpected patterns in the results, particularly the relatively low spelling accuracy and phonological awareness performance by the alphabetic L1 speakers. To some degree, these unexpected findings can be accounted for when considering another way to analyze the data: in terms of error rates, rather than strict accuracy. This highlights perhaps the most important individual finding from the study: the substantially different patterns of results, and conclusions, that may be reached when the data are scored differently. The field of L2 literacy research has much important work to do to document such variations and establish best practices for task choice, item selection, and particularly scoring procedures to improve reliability and validity.

## Data Availability Statement

The datasets generated for this study are available on request to the corresponding author.

## Ethics Statement

The studies involving human participants were reviewed and approved by Human Subjects Committee at Southern Illinois University Carbondale. The patients/participants provided their written informed consent to participate in this study.

## Author Contributions

KM contributed to study design, item selection, data collection, data analysis, and writing. EL contributed to data analysis and writing. KC contributed to study design, item selection, data collection, and writing. EH contributed to study design, item selection, data collection, and writing.

## Conflict of Interest

The authors declare that the research was conducted in the absence of any commercial or financial relationships that could be construed as a potential conflict of interest.

## Footnotes

1 Both feedback (spelling) and feed-forward (reading) consistency information was obtained from Ziegler et al. (1997), giving each syllable two consistency judgments. For words with more than one syllable, feedback and feed-forward consistency were determined for each syllable separately. This was done as a proxy for consistency in multisyllabic words, given that objective statistics for the consistency of such words are not available.

## References

[B1] Abu-Rabia S. (2001). The role of vowels in reading semitic scripts: data from Arabic and Hebrew. *Read. Writ.* 14 39–59. 10.1023/a:1008147606320

[B2] Abu-Rabia S. (2002). Reading in a root–based–morphology language: the case of Arabic. *J. Res. Read.* 25 299–309. 10.1111/1467-9817.00177

[B3] Adams M. J. (1990). *Begnning to Read: Thinking and Learning About Print.* Cambridge, MA: MIT Press.

[B4] Akamatsu N. (2003). The effects of first language orthographic features on second language reading in text. *Lang. Learn.* 53 207–231. 10.1111/1467-9922.00216

[B5] Allaith Z. A. Joshi R. M. (2011). Spelling performance of English consonants among students whose first language is Arabic. *Read. Writ.* 24 1089–1110. 10.1007/s11145-010-9294-3

[B6] Anthony J. L. Lonigan C. J. Driscoll K. Phillips B. M. Burgess S. R. (2003). Phonological sensitivity: a quasi-parallel progression of word structure units and cognitive operations. *Read. Res. Q.* 38 470–487. 10.1598/RRQ.38.4.3

[B7] Anthony J. L. Williams J. M. Durán L. K. Gillam S. L. Liang L. Aghara R. (2011). Spanish phonological awareness: dimensionality and sequence of development during the preschool and kindergarten years. *J. Educ. Psychol.* 103 857–876. 10.1037/a0025024

[B8] Apel K. (2011). What is orthographic knowledge? *Lang. SpeechHear. Services Sch.* 42 592–603. 10.1044/0161-1461(2011/10-0085) 21844399

[B9] Apel K. Henbest V. S. Masterson J. (2019). Orthographic knowledge: clarifications, challenges, and future directions. *Read. Writ.* 32 873–889. 10.1007/s11145-018-9895-9

[B10] Arndt E. J. Foorman B. R. (2010). Second graders as spellers: what types of errors are they making? *Assess. Effect. Intervent.* 36 57–67. 10.1177/1534508410380135

[B11] Baron J. (1979). Orthographic and word-specific mechanisms in children’s reading of words. *Child Dev.* 50 60–72. 10.2307/1129042

[B12] Bates D. Mächler M. Bolker B. Walker S. (2015). Fitting linear mixed-effects models using lme4. *J. Stat. Softw.* 67 1–48. 10.18637/jss.v067.i01

[B13] Bear D. R. Invernizzi M. Templeton S. Johnston F. (2019). *Words Their Way: Word Study for Phonics, Vocabulary, and Spelling Instruction.* Hoboken, NJ: Pearson Education.

[B14] Berninger V. W. Amtmann D. (2003). “Preventing written expression disabilities through early and continuing assessment and intervention for handwriting and/or spelling problems: research into practice,” in *Handbook of Learning Disabilities*, eds Swanson H. L. Harris K. R. Graham S. (New York, NY: Guilford Press), 345–363.

[B15] Berninger V. W. Vaughan K. Abbott R. D. Begay K. Coleman K. B. Curtin G. (2002). Teaching spelling and composition alone and together: implications for the simple view of writing. *J. Educ. Psychol.* 94 291–304. 10.1037/0022-0663.94.2.291

[B16] Branum-Martin L. Tao S. Garnaat S. Bunta F. Francis D. J. (2012). Meta-analysis of bilingual phonological awareness: language, age, and psycholinguistic grain size. *J. Educ. Psychol.* 104 932–944. 10.1037/a0027755

[B17] Bruck M. Treiman R. (1990). Phonological awareness and spelling in normal children and dyslexics: the case of initial consonant clusters. *J. Exp. Child Psychol.* 50 156–178. 10.1016/0022-0965(90)90037-9 2398331

[B18] Burgess S. R. Lonigan C. J. (1998). Bidirectional relations of phonological sensitivity and prereading abilities: evidence from a preschool sample. *J. Exp. Child Psychol.* 70 117–141. 10.1006/jecp.1998.2450 9729452

[B19] Cassar M. Treiman R. (2004). “Developmental variations in spelling: comparing typical and poor spellers,” in *Handbook of Language and Literacy: Development and Disorders*, eds Stone C. A. Silliman E. R. Ehren B. J. Apel K. (New York, NY: Guilford), 627–643.

[B20] Castles A. Holmes V. M. Wong M. (1997). Variations in spelling style among lexical and sublexical readers. *J. Exp. Child Psychol.* 64 98–118. 10.1006/jecp.1996.2339

[B21] Chall J. S. (1983). *Stages of Reading Development.* New York, NY: McGraw-Hill.

[B22] Chao Y. R. (1968). *Language and Symbolic Systems.* New York, NY: Cambridge University Press.

[B23] Clemens N. H. Oslund E. L. Simmons L. E. Simmons D. (2014). Assessing spelling in kindergarten: further comparison of scoring metrics and their relation to reading skills. *J. Sch. Psychol.* 52 49–61. 10.1016/j.jsp.2013.12.005 24495494

[B24] Coltheart M. Curtis B. Atkins P. Haller M. (1993). Models of reading aloud: dual-route and parallel-distributed-processing approaches. *Psychol. Rev.* 100 589–608. 10.1037/0033-295X.100.4.589

[B25] Coltheart M. Rastle K. Perry C. Langdon R. Ziegler J. C. (2001). DRC: a dual route cascaded model of visual word recognition and reading aloud. *Psychol. Rev.* 108 204–256. 10.1037/0033-295x.108.1.204 11212628

[B26] Conrad N. J. Harris N. Williams J. (2013). Individual differences in children’s literacy development: the contribution of orthographic knowledge. *Read. Writ.* 26 1223–1239. 10.1007/s11145-012-9415-2

[B27] Cook V. J. (1997). L2 users and English spelling. *J. Multiling. Multicult. Dev.* 18 474–488. 10.1080/01434639708666335

[B28] Council of Europe (2001). *Common European Framework of Reference for Languages: Learning, Teaching, Assessment.* Cambridge: Cambridge University Press.

[B29] Cronnell B. (1985). Language influences in the English writing of third- and sixth-grade Mexican-American students. *J. Educ. Res.* 78 168–173. 10.1080/00220671.1985.10885594

[B30] Crystal D. (2003). *English as a Global Language.* New York, NY: Cambridge University Press.

[B31] Cunningham A. E. Perry K. E. Stanovich K. E. (2001). Converging evidence for the concept of orthographic processing. *Read. Writ.* 14 549–568. 10.1023/A:1011100226798

[B32] Daniels P. T. Bright W. (1996). *The World’s Writing Systems.* New York, NY: Oxford University Press.

[B33] de Manrique A. M. B. Signorini A. (1994). Phonological awareness, spelling and reading abilities in Spanish-speaking children. *Br. J. Educ. Psychol.* 64 429–439. 10.1111/j.2044-8279.1994.tb01114.x

[B34] Deacon S. H. Benere J. Castles A. (2012). Chicken or egg? Untangling the relationship between orthographic processing skill and reading accuracy. *Cognition* 122 110–117. 10.1016/j.cognition.2011.09.003 22030120

[B35] Deacon S. H. Conrad N. Pacton S. (2008). A statistical learning perspective on children’s learning about graphotactic and morphological regularities in spelling. *Can. Psychol. Psychol. Can.* 49 118.

[B36] DeFrancis J. (1989). *Visible Speech: The Diverse Oneness of Writing Systems.* Honolulu, HI: University of Hawaii Press.

[B37] Dich N. Pedersen B. (2013). Native language effects on spelling in English as a foreign language: a time-course analysis. *Can. J. Appl. Linguist. Rev. Can. Linguist. Appl.* 16 51–68.

[B38] Dixon L. Q. Zhao J. Joshi R. M. (2010). Influence of L1 orthography on spelling English words by bilingual children - A natural experiment comparing syllabic, phonological, and morphosyllabic first languages. *Learn. Disabi. Q.* 33 211–221.

[B39] Durgunoğlu A. Y. (1998). “Acquiring literacy in English and Spanish in the United States,” in *Literacy Development in a Multilingual Context: Cross-Cultural Perspectives*, eds Durgunoğlu A. Y. Verhoeven L. (Mahwah, NJ: Erlbaum), 135–145.

[B40] Durgunoğlu A. Y. (2002). Cross-linguistic transfer in literacy development and implications for language learners. *Ann. Dyslexia* 52 189–204. 10.1007/s11881-002-0012-y

[B41] Ehri L. C. (1989). The development of spelling knowledge and its role in reading acquisition and reading disability. *J. Learn. Disabil.* 22 356–365. 10.1177/002221948902200606 2738469

[B42] Ehri L. C. (1997). “Learning to read and learning to spell are one and the same, almost,” in *Learning to Spell: Research, Theory, and Practice Across Languages*, eds Perfetti C. A. Rieben L. Fayol M. (Mahwah, NJ: Lawrence Erlbaum Associates), 237–269.

[B43] Ehri L. C. (2000). Learning to read and learning to spell: two sides of a coin. *Top. Lang. Disord.* 20 19–36.

[B44] Ehri L. C. (2005). Learning to read words: theory, findings, and issues. *Sci. Stud. Read.* 9 167–188. 10.1207/s1532799xssr0902_4

[B45] Ehri L. C. Snowling M. J. (2004). “Developmental variation in word recognition,” in *Handbook of Language and Literacy: Development and Disorders*, eds Stone C. A. Silliman E. R. Ehren B. J. Apel K. (New York, NY: Guilford Press), 433–460.

[B46] Ehri L. C. Wilce L. S. (1987). Does learning to spell help beginners learn to read words? *Read Res. Q.* 22 47–65. 10.2307/747720

[B47] Fashola O. S. Drum P. A. Mayer R. E. Kang S.-J. (1996). A cognitive theory of orthographic transitioning: predictable errors in how Spanish-speaking children spell English words. *Am. Educ. Res. J.* 33 825–843. 10.3102/00028312033004825

[B48] Fender M. (2003). English word recognition and word integration skills of native Arabic- and Japanese-speaking learners of English as a second language. *Appl. Psycholinguist.* 24 289–315. 10.1017/S014271640300016X

[B49] Fender M. (2008). Spelling knowledge and reading development: insights from Arab ESL learners. *Read. Foreign. Lang.* 20 19–42.

[B50] Ferroli L. Shanahan T. (1992). “Voicing in Spanish to English spelling knowledge transfer,” in *Proceeding of the 42nd Annual Meeting of the National Reading Conference*, San Antonio, TX.

[B51] Figueredo L. (2006). Using the known to chart the unknown: a review of first-language influence on the development of English-as-a-second-language spelling skill. *Read. Writ.* 19 873–905. 10.1007/s11145-006-9014-1

[B52] Frith U. (1985). “Beneath the surface of developmental dyslexia,” in *Surface Dyslexia: Neuropsychological and Cognitive Studies of Phonological Reading*, eds Patterson K. Marshall J. Coltheart M. (London: Lawrence Erlbaum Associates), 301–330.

[B53] Frith U. Wimmer H. Landerl K. (1998). Differences in phonological recoding in German- and English-speaking children. *Sci. Stud. Read.* 2 31–54. 10.1207/s1532799xssr0201_2

[B54] Frost R. (2006). Becoming literate in Hebrew: the grain size hypothesis and Semitic orthographic systems. *Dev. Sci.* 9 439–440. 10.1111/j.1467-7687.2006.00523.x 16911440

[B55] Frost R. (2012). Towards a universal model of reading. *Behav. Brain Sci.* 35 263–279. 10.1017/S0140525X12001574 22929057 PMC3677812

[B56] Georgiou G. K. Parrila R. Kirby J. R. (2009). RAN components and reading development from grade 3 to grade 5: what underlies their relationship? *Sci. Stud. Read.* 13 508–534. 10.1080/10888430903034796

[B57] Gill J. T. (1989). The relationship between word recognition and spelling in the primary grades. *Read. Psychol.* 10 117–136. 10.1080/0270271890100202

[B58] Gomez L. M. Gomez K. (2007). Reading for learning: literacy supports for 21st-century work. *Phi Delta Kappan* 89 224–228.

[B59] Goswami U. Bryant P. E. (1990). *Phonological Skills and Learning to Read.* Hove: Erlbaum. 10.1111/j.1749-6632.1993.tb22977.x 8323121

[B60] Goswami U. Gombert J. E. de Barrera L. F. (1998). Children’s orthographic representations and linguistic transparency: nonsense word reading in English, French, and Spanish. *Appl. Psycholinguist.* 19 19–52. 10.1017/S0142716400010560

[B61] Grabe W. (2009). *Reading in a Second Language: Moving From Theory to Practice.* Cambridge: Cambridge University Press.

[B62] Graham S. (1999). Handwriting and spelling instruction for students with learning disabilities: a review. *Learn. Disabil. Q.* 22 78–98. 10.2307/1511268

[B63] Graham S. Berninger V. W. Abbott R. D. Abbott S. P. Whitaker D. (1997). Role of mechanics in composing of elementary school students: a new methodological approach. *J. Educ. Psychol.* 89 170–182.

[B64] Graham S. Harris K. R. Chorzempa B. F. (2002). Contribution of spelling instruction to the spelling, writing, and reading of poor spellers. *J. Educ. Psychol.* 94 669–686.

[B65] Graham S. Hebert M. (2011). Writing to read: a meta-analysis of the impact of writing and writing instruction on reading. *Harvard Educ. Rev.* 81 710–744. 10.17763/haer.81.4.t2k0m13756113566

[B66] Graham S. Santangelo T. (2014). Does spelling instruction make students better spellers, readers, and writers? A meta-analytic review. *Read. Writ.* 27 1703–1743. 10.1007/s11145-014-9517-0

[B67] Grant A. Gottardo A. Geva E. (2012). Measures of reading comprehension: do they measure different skills for children learning English as a second language? *Read. Writ.* 25 1899–1928. 10.1007/s11145-012-9370-y

[B68] Greenberg D. Ehri L. C. Perin D. (1997). Are word-reading processes the same or different in adult literacy students and third–fifth graders matched for reading level? *J. Educ. Psychol.* 89 262–275. 10.1037/0022-0663.89.2.262

[B69] Greenberg D. Ehri L. C. Perin D. (2002). Do adult literacy students make the same word-reading and spelling errors as children matched for word-reading age? *Sci. Stud. Read.* 6 221–243. 10.1207/S1532799XSSR0603_2

[B70] Greenberg D. Pae H. K. Morris R. D. Calhoon M. B. Nanda A. O. (2009). Measuring adult literacy students’ reading skills using the Gray Oral Reading Test. *Ann. Dyslexia* 59 133–149. 10.1007/s11881-009-0027-8 19629705 PMC2794989

[B71] Haggan M. (1993). Actual and self-perceived spelling accuracy in Kuwaiti EFL students: some practical and theoretical implications. *TESL Can. J.* 10 55–70.

[B72] Hamada M. Koda K. (2008). Influence of first language orthographic experience on second language decoding and word learning. *Lang. Learn.* 58 1–31. 10.1111/j.1467-9922.2007.00433.x

[B73] Hanley R. Masterson J. Spencer L. Evans D. (2004). How long do the advantages of learning to read a transparent orthography last? An investigation of the reading skills and reading impairment of Welsh children at 10 years of age. *Q. J. Exp. Psychol. Section A* 57 1393–1410. 10.1080/02724980343000819 15513252

[B74] Hayes-Harb R. (2006). Native speakers of Arabic and ESL texts: evidence for the transfer of written word identification processes. *TESOL Q.* 40 321–339.

[B75] Helman L. Bear D. R. Templeton S. Invernizzi M. A. Johnston F. (2012). *Words Their Way With English Learners: Word Study for Phonics, Vocabulary, and Spelling.* Upper Saddle River, NJ: Pearson Education.

[B76] Holm A. Dodd B. (1996). The effect of first written language on the acquisition of English literacy. *Cognition* 59 119–147. 10.1016/0010-0277(95)00691-5 8681508

[B77] Huang H. S. Hanley J. R. (1995). Phonological awareness and visual skills in learning to read Chinese and English. *Cognition* 54 73–98. 10.1016/0010-0277(94)00641-W 7851080

[B78] Hudson T. (2007). *Teaching Second Language Reading.* Oxford: Oxford University Press.

[B79] Ibrahim M. H. (1978). Patterns in spelling errors. *ELT J.* 32 207–212. 10.1093/elt/XXXII.3.207

[B80] James C. Klein K. (1994). Foreign language learners’ spelling and proofreading strategies. *Papers Stud. Contrast. Linguist.* 29 31–46.

[B81] Jongejan W. Verhoeven L. Siegel L. S. (2007). Predictors of reading and spelling abilities in first-and second-language learners. *J. Educ. Psychol.* 99 835–851.

[B82] Joshi R. M. Treiman R. Carreker S. Moats L. C. (2008). How words cast their spell. *Am. Educ.* 32 6–16.

[B83] Juffs A. Martin K. I. (2014). “Impacts of L1 orthography and item characteristics on L2 spelling knowledge,” in *Second Language Research Forum*, (Charleston, SC: University of South Carolina).

[B84] Koda K. (2004). *Insights Into Second Language Reading: A Cross-Linguistic Approach.* New York, NY: Cambridge University Press.

[B85] Koda K. (2008). “Impacts of prior literacy experience on second-language learning to read,” in *Learning to Read Across Languages*, eds Koda K. Zehler A. M. (New York, NY: Routledge), 68–96.

[B86] Koda K. Zehler A. M. (2008). *Learning to Read Across Languages: Cross-Linguistic Relationships in First- and Second-Language Literacy Development.* New York, NY: Routledge.

[B87] Kuperman V. Bertram R. Baayen R. H. (2008). Morphological dynamics in compound processing. *Lang. Cogn. Process.* 23 1089–1132. 10.1080/01690960802193688

[B88] Lennox C. Siegel L. S. (1993). Visual and phonological spelling errors in subtypes of children with learning disabilities. *Appl. Psycholinguist.* 14 473–488. 10.1017/S0142716400010705

[B89] Lennox C. Siegel L. S. (1996). The development of phonological rules and visual strategies in average and poor spellers. *J. Exp. Child Psychol.* 62 60–83. 10.1006/jecp.1996.0022 8683185

[B90] Lennox C. Siegel L. S. (1998). “Phonological and orthographic processes in good and poor spellers,” in *Reading and Spelling: Development and Disorders*, eds Hulme C. Joshi R. M. (Mahwah, NJ: Lawrence Erlbaum Associates), 395–404.

[B91] Leong C. (2011). “Chinese language learners of English use more orthographic-lexical than phonological strategies in English word recognition and spelling,” in *Language and Literacy Development in Bilingual Settings*, eds Durgunoğlu A. Y. Goldenberg C. (New York, NY: The Guilford Press), 188–209.

[B92] Li N. H. Martin K. I. (2017). “Orthographical errors in beginning and intermediate learners of L2 Japanese from two L1s,” in *Proceedings of the 2016 Pacific Second Language Research Forum*, 127–132.

[B93] Liow S. J. R. Poon K. K. L. (1998). Phonological awareness in multilingual Chinese children. *Appl. Psycholinguist.* 19 339–362. 10.1017/S0142716400010213

[B94] Loveall S. J. Channell M. M. Phillips B. A. Conners F. A. (2013). Phonological recoding, rapid automatized naming, and orthographic knowledge. *J. Exp. Child Psychol.* 116 738–746. 10.1016/j.jecp.2013.05.009 23827643

[B95] MacDonald G. W. Cornwall A. (1995). The relationship between phonological awareness and reading and spelling achievement eleven years later. *J. Learn. Disabil.* 28 523–527. 10.1177/002221949502800807 7595043

[B96] Martin K. I. (2017). The impact of L1 writing system on ESL knowledge of vowel and consonant spellings. *Read. Writ.* 30 279–298. 10.1007/s11145-016-9673-5

[B97] Martin K. I. Juffs A. (in press). *Eye-Tracking as a Window into Assembled Phonology in Native and Non-native Reading.* University of Southern Illinois, University of Pittsburgh Pittsburgh, PA.

[B98] Martin K. I. Li N. H. (2017). “Overlap in functional and orthographic written errors by L2 learners of Japanese,” in *Proceedings of the 23rd Princeton Japanese Pedagogy Forum*, Princeton, NJ, 234–259.

[B99] Masterson J. Apel K. (2007). “Spelling and word-level reading: a multilinguistic approach,” in *Clinical Decision Making in Developmental Language Disorders*, eds Kamhi A. G. Masterson J. Apel K. (Baltimore, MD: Paul H. Brookes Publishing Company), 249–266.

[B100] Masterson J. J. Apel K. (2010). The spelling sensitivity score: noting developmental changes in spelling knowledge. *Assess. Effect. Intervent.* 36 35–45. 10.1177/1534508410380039

[B101] Mayer P. Crowley K. Kaminska Z. (2007). Reading and spelling processes in Welsh–English bilinguals: differential effects of concurrent vocalisation tasks. *Read. Writ.* 20 671–690. 10.1007/s11145-006-9044-8

[B102] McBride-Chang C. (1995). What is phonological awareness? *J. Educ. Psychol.* 87 179–192. 10.1037/0022-0663.87.2.179

[B103] McBride-Chang C. Bialystok E. Chong K. K. Y. Li Y. (2004). Levels of phonological awareness in three cultures. *J. Exp. Child Psychol.* 89 93–111. 10.1016/j.jecp.2004.05.001 15388300

[B104] Mehta P. D. Foorman B. R. Branum-Martin L. Taylor W. P. (2005). Literacy as a unidimensional multilevel construct: validation, sources of influence, and implications in a longitudinal study in grades 1 to 4. *Sci. Stud. Read.* 9 85–116. 10.1207/s1532799xssr0902_1

[B105] Moats L. C. (2005). How spelling supports reading. *Am. Educ.* 6 42–43.

[B106] Moats L. C. Foorman B. Taylor P. (2006). How quality of writing instruction impacts high-risk fourth graders’ writing. *Read. Writ.* 19 363–391. 10.1007/s11145-005-4944-6

[B107] Nanda A. O. Greenberg D. Morris R. D. (2014). Reliability and validity of the CTOPP Elision and Blending Words subtests for struggling adult readers. *Read. Writ.* 27 1603–1618. 10.1007/s11145-014-9509-0

[B108] Nassaji H. Geva E. (1999). The contribution of phonological and orthographic processing skills to adult ESL reading: evidence from native speakers of Farsi. *Appl. Psycholinguist.* 20 241–267.

[B109] Nunes T. Bryant P. Bindman M. (1997). Morphological spelling strategies: developmental stages and processes. *Dev. Psychol.* 33 637–649. 10.1037/0012-1649.33.4.637 9232379

[B110] Pae H. K. Greenberg D. Williams R. S. (2012). An analysis of differential response patterns on the Peabody Picture Vocabulary Test-IIIB in struggling adult readers and third-grade children. *Read. Writ.* 25 1239–1258. 10.1007/s11145-011-9315-x

[B111] Perfetti C. A. (2007). Reading ability: lexical quality to comprehension. *Sci. Stud. Read.* 11 357–383. 10.1080/10888430701530730

[B112] Perfetti C. A. Beck I. Bell L. C. Hughes C. (1987). Phonemic knowledge and learning to read are reciprocal: a longitudinal study of first grade children. *Merrill Palmer Q.* 33 283–319.

[B113] Perfetti C. A. Dunlap S. (2008). “Learning to read: general principles and writing system variations,” in *Learning to Read Across Languages: Cross-Linguistic Relationships in First- and Second-Language Literacy Development*, eds Koda K. Zehler A. M. (New York, NY: Routledge), 13–38.

[B114] Perfetti C. A. Hart L. (2002). “The lexical quality hypothesis,” in *Precursors of Functional Literacy*, eds Verhoeven L. Elbro C. Reitsma P. (Philadelphia, PA: John Benjamins), 189–213.

[B115] Perfetti C. A. Stafura J. (2014). Word knowledge in a theory of reading comprehension. *Sci. Stud. Readi.* 18 22–37. 10.1080/10888438.2013.827687

[B116] Perfetti C. A. Verhoeven L. (eds) (2017). *Learning to Read Across Languages and Writing Systems.* Cambridge: Cambridge University Press.

[B117] Plaut D. C. McClelland J. L. Seidenberg M. S. Patterson K. (1996). Understanding normal and impaired word reading: computational principles in quasi-regular domains. *Psychol. Rev.* 103 56–115. 8650300 10.1037/0033-295x.103.1.56

[B118] Post Y. V. Carreker S. Holland G. (2001). The spelling of final letter patterns: a comparison of instruction at the level of the phoneme and the rime. *Ann. Dyslexia* 51 121–146. 10.1007/s11881-001-0008-z

[B119] Randall M. Meara P. (1988). How Arabs read Roman letters. *Read. a Foreign Lang.* 4 133–145.

[B120] Re A. M. Pedron M. Cornoldi C. (2007). Expressive writing difficulties in children described as exhibiting ADHD symptoms. *J. Learn. Disabil.* 40 244–255. 10.1177/00222194070400030501 17518216

[B121] Read C. Zhang Y.-F. Nie H.-Y. Ding B.-Q. (1986). The ability to manipulate speech sounds depends on knowing alphabetic writing. *Cognition* 24 31–44. 10.1016/0010-0277(86)90003-X 3791920

[B122] Russak S. Kahn-Horwitz J. (2015). English as a foreign language spelling: comparisons between good and poor spellers. *J. Res. Read.* 38 307–330. 10.1111/jrir.12009

[B123] Ryan A. Meara P. (1991). The case of the invisible vowels: Arabic speakers reading English words. *Read. a Foreign Lang.* 7 531–540.

[B124] Ryan A. Meara P. (1996). “A diagnostic test for vowel blindness in Arabic speaking learners of English,” in *The _Lognostics Virtual Library*, (Sketty: Swansea University).

[B125] Saigh K. Schmitt N. (2012). Difficulties with vocabulary word form: the case of Arabic ESL learners. *System* 40 24–36. 10.1016/j.system.2012.01.005

[B126] Sampson G. (2015). *Writing systems.* Bristol, CT: Equinox.

[B127] San Francisco A. R. Mo E. Carlo M. August D. Snow C. (2006). The influences of language of literacy instruction and vocabulary on the spelling of Spanish–English bilinguals. *Read. Writ.* 19 627–642. 10.1007/s11145-006-9012-3

[B128] Seidenberg M. S. McClelland J. L. (1989). A distributed, developmental model of word recognition and naming. *Psychol. Rev.* 96 523–568. 10.1037/0033-295X.96.4.523 2798649

[B129] Share D. L. (2008). On the Anglocentricities of current reading research and practice: the perils of overreliance on an “outlier” orthography. *Psychol. Bull.* 134 584–615. 10.1037/0033-2909.134.4.584 18605821

[B130] Share D. L. Levin I. (1999). “Learning to read and write in Hebrew,” in *Learning to Read and Write: A Cross-Linguistic Perspective*, eds Harris M. Hatano G. (Cambridge: Cambridge University Press), 89–111.

[B131] Singer B. D. Bashir A. S. (2004). “Developmental variations in writing composition skills,” in *Handbook of Language and Literacy: Development and Disorders*, eds Stone C. A. Silliman E. R. Ehren B. J. Apel K. (New York, NY: Guilford Press), 559–582.

[B132] Snow C. Griffin P. Burns M. S. (2005). *Knowledge to Support the Teaching of Reading: Preparing Teachers for a Changing World.* San Francisco, CA: John Wiley & Sons.

[B133] Snow C. E. Strucker J. (1999). “Lessons from preventing reading difficulties in young children for adult learning and literacy,” in *Office of Educational Research and Improvement*, eds Comings J. Garner B. Smith C. (San Francisco, CA: Jossey-Bass), 25–73.

[B134] Sun-Alperin M. K. Wang M. (2008). Spanish-speaking children’s spelling errors with English vowel sounds that are represented by different graphemes in English and Spanish words. *Contemp. Educ. Psychol.* 33 932–948. 10.1016/j.cedpsych.2007.12.005

[B135] Tolchinsky L. Levin I. Aram D. McBride-Chang C. (2012). Building literacy in alphabetic, abjad and morphosyllabic systems. *Read. Writ.* 25 1573–1598. 10.1007/s11145-011-9334-7

[B136] Treiman R. (1984). Individual differences among children in spelling and reading styles. *J. Exp. Child Psychol.* 37 463–477. 10.1016/0022-0965(84)90071-7 6747544

[B137] Treiman R. (1997). “Introduction to special issue on spelling,” in *Spelling*, ed. Treiman R. (Dordrecht: Springer), 1–5.

[B138] Verhoeven L. Schreuder R. Baayen R. H. (2006). Learnability of graphotactic rules in visual word identification. *Learn. Instruc.* 16 538–548. 10.1016/j.learninstruc.2006.10.003

[B139] Wade-Woolley L. (1999). First language influences on second language word reading: all roads lead to Rome. *Lang. Learn.* 49 447–471. 10.1111/0023-8333.00096

[B140] Wade-Woolley L. Siegel L. S. (1997). “The spelling performance of ESL and native speakers of English as a function of reading skill,” in *Spelling*, ed. Treiman R. (Dordrecht: Springer), 73–92.

[B141] Wagner R. K. Torgesen J. K. Rashotte C. A. (1999). *Comprehensive Test of Phonological Processing (CTOPP).* Austin, TX: Pro-Ed.

[B142] Wang F. Tsai Y. Wang W. S.-Y. (2009). “Chinese literacy,” in *Cambridge Handbook on Literacy*, eds Olson D. R. Torrance N. (New York, NY: Cambridge University Press).

[B143] Wang M. Geva E. (2003a). Spelling acquisition of novel English phonemes in Chinese children. *Read. Writ.* 16 325–348. 10.1023/a:1023661927929

[B144] Wang M. Geva E. (2003b). Spelling performance of Chinese children using English as a second language: lexical and visual–orthographic processes. *Appl. Psycholinguist.* 24 1–25. 10.1017/S0142716403000018

[B145] Wang M. Koda K. (2005). Commonalities and differences in word identification skills among learners of English as a second language. *Lang. Learn.* 55 71–98. 10.1111/j.0023-8333.2005.00290.x

[B146] Wang M. Koda K. (2007). Commonalities and differences in word identification skills among learners of English as a second language. *Lang. Learn.* 57(Suppl.1) 201–222. 10.1111/j.1467-9922.2007.00416.x

[B147] Wang M. Koda K. Perfetti C. A. (2003). Alphabetic and nonalphabetic L1 effects in English word identification: a comparison of Korean and Chinese English L2 learners. *Cognition* 87 129–149. 10.1016/s0010-0277(02)00232-9 12590041

[B148] Winke P. Lee S. Ahn J. I. Choi I. Cui Y. Yoon H.-J. (2018). The cognitive validity of child English language tests: what young language learners and their native-speaking peers can reveal. *TESOL Q.* 52 274–303. 10.1002/tesq.396

[B149] Wollscheid S. Sjaastad J. Tømte C. (2016). The impact of digital devices vs. pen(cil) and paper on primary school students’ writing skills – A research review. *Comput. Educ.* 95 19–35. 10.1016/j.compedu.2015.12.001

[B150] Yeung P.-S. Ho C. S.-H. Chik P. P.-M. Lo L.-Y. Luan H. Chan D. W.-O. (2011). Reading and spelling Chinese among beginning readers: what skills make a difference? *Sci. Stud. Read.* 15 285–313. 10.1080/10888438.2010.482149

[B151] Ziegler J. C. Goswami U. (2005). Reading acquisition, developmental dyslexia, and skilled reading across languages: a psycholinguistic grain size theory. *Psychol. Bull.* 131 3–29. 10.1037/0033-2909.131.1.3 15631549

[B152] Ziegler J. C. Stone G. Jacobs A. (1997). What is the pronunciation for -ough and the spelling for/u/? A database for computing feedforward and feedback consistency in English. *Behav. Res. MethodsInstrum. Comput.* 29 600–618. 10.3758/bf03210615

[B153] Zutell J. Allen V. (1988). The English spelling strategies of Spanish-speaking bilingual children. *TESOL Q.* 22 333–340. 10.2307/3586941

