# Supplementary material for: English Word and Pseudoword Spellings and Phonological Awareness: Detailed Comparisons From Three L1 Writing Systems
Source: Front Psychol. 2020 Jul 2;11:1309. doi: 10.3389/fpsyg.2020.01309 (PMC7343770; doi:10.3389/fpsyg.2020.01309)
Supplement: Supplementary file 1 [file Table_1.DOCX]

**Supplementary Materials**

**Real Word Spelling Items**

cook

dress

train

watch

share

choice

catch

found

strange

burn

throw

grown

music

customer

separate

babies

written

napkin

swimming

success

surprise

purchase

kitchen

suggestion

distance

decision

recognize

necessary

electrical

responsible

furniture

preparation

interesting

knowledge

Lexical characteristics:

|  | Mean | SD |
| --- | --- | --- |
| Length (letters) | 7.18 | 2.10 |
| Length (phonemes) | 5.62 | 2.10 |
| Length (syllables) | 2.12 | 1.07 |
| Length (morphemes) | 1.50 | .56 |
| Log frequency^a^ | 10.10 | 1.18 |
| Number of orthographic neighbors^b^ | 3.56 | 4.87 |
| Frequency of orthographic neighbors^b^ | 15.61 | 28.05 |
| Number of phonological neighbors^b^ | 7.91 | 11.10 |
| Frequency of phonological neighbors^b^ | 35.62 | 71.09 |
| Bigram Sum^c^ | 26836.53 | 18200.82 |
| Bigram Mean^c^ | 4108.29 | 1671.34 |
| Concreteness^c^ | 3.29 | 1.15 |
| Age of Acquisition^c^ | 6.10 | 1.96 |
| Number of feedback consistent syllables^d^ | 27 | |
| Number of feedback inconsistent syllables^d^ | 45 | |
| Number of feed-forward consistent syllables^d^ | 44 | |
| Number of feed-forward inconsistent syllables^d^ | 28 | |

*Note.* ^a^Frequency statistics are from the HAL frequencies available from E-Lexicon (Balota et al., 2007). ^b^From CLEARPOND (Marian et al., 2012). ^c^From E-Lexicon (Balota et al., 2007). ^d^Calculated from Ziegler, Stone and Jacobs (1997).

**Pseudoword Spelling Items**

swead /swɛd/

spood /spud/

bave /beɪv/

chook /tʃʊk/

shome /ʃoʊm/

kep /kɛp/

tuss /tʌs/

nesh /nɛʃ/

vist /vɪst/

thop /θɑp/

prand /pɹænd/

fint /fɪnt/

bap /bæp/

cleal /klil/

stull /stʌl/

hine /haɪn/

Lexical characteristics:

|  | Mean | SD |
| --- | --- | --- |
| Length (letters) | 4.31 | .70 |
| Length (phonemes) | 3.5 | .63 |
| Length (syllables) | 1 | 0 |
| Number of orthographic neighbors^a^ | 9 | 6.67 |
| Frequency of orthographic neighbors^a^ | 100.61 | 125.93 |
| Positional Biphone Frequency^a^ | .005 | .004 |
| Positional Letter Frequency^a^ | .05 | .01 |
| Number of feedback consistent syllables^b^ | 7 | |
| Number of feedback inconsistent syllables^b^ | 9 | |
| Number of feed-forward consistent syllables^b^ | 7 | |
| Number of feed-forward inconsistent syllables^b^ | 9 | |

*Note.* ^a^From CLEARPOND (Marian et al., 2012). ^b^Calculated from Ziegler, Stone and Jacobs (1997).
